# NUDT16 enhances the resistance of cancer cells to DNA-damaging agents by regulating replication fork stability *via* reversing HMGA1 ADP-ribosylation

**DOI:** 10.1016/j.jbc.2025.108551

**Published:** 2025-04-25

**Authors:** Yingshi Zhou, Zhihuai Deng, Shiyu Xiong, Wenjia Li, Wanrong Luo, Man Luo, Haifeng Tang, Wenjing Wu, Carmen Chak-Lui Wong, Dong Yin, Kaishun Hu, Baoming Luo

**Affiliations:** 1 Department of Ultrasound, Sun Yat-Sen Memorial Hospital, Sun Yat-Sen University, Guangzhou, China; 2 Guangdong Provincial Key Laboratory of Malignant Tumor Epigenetics and Gene Regulation, Guangdong-Hong Kong Joint Laboratory for RNA Medicine, Medical Research Center, Sun Yat-Sen Memorial Hospital, Sun Yat-Sen University, Guangzhou, China; 3 Department of Spine Surgery, Sun Yat-sen Memorial Hospital of Sun Yat-sen University, Guangzhou, China; 4 Department of Pathology, The First Affiliated Hospital, Zhengzhou University, Zhengzhou, China; 5 Department of Breast Oncology, Sun Yat-Sen Memorial Hospital, Sun Yat-Sen University, Guangzhou, China; 6 Li Ka Shing Faculty of Medicine, Department of Pathology, The University of Hong Kong, Hong Kong, China

**Keywords:** HMGA1, NUDT16, replication stress, PARylation, ubiquitination

## Abstract

Precise DNA replication is the basis for maintaining cell proliferation and genome stability. Current chemotherapy drugs and radiotherapy induce cell death by aggravating replication stress, albeit with poor efficacy. The replication stress response has been shown to play fundamental roles in resistance to radiotherapy and chemotherapy. High mobility group A1 (HMGA1) promotes tumor progression by regulating autophagy, angiogenesis, and chemoresistance; however, its role in coordinating replication stress and cell cycle progression remains elusive. Our results indicated that HMGA1 recruited FANCD2 to promote DNA replication and cell cycle progression both by attenuating R-loop-induced replication stress and by protecting stalled replication forks from degradation, ultimately enhancing tumor resistance to chemotherapy and irradiation treatment. We also identified HMGA1 as a novel substrate for the dePARylase NUDT16. NUDT16 was found to suppress the binding of HMGA1 to the E3 ubiquitin ligase CHFR by removing its PARylation at Glu 50, thereby reducing its ubiquitin-proteasome pathway-mediated degradation and enhancing HMGA1 protein stability. NUDT16-HMGA1 inhibition can significantly improve the sensitivity of tumor cells to chemotherapy and irradiation treatment. Collectively, these data suggest that NUDT16 enhances the ability of tumor cells to cope with replication stress by reversing the PARylation and positively regulating the protein expression of HMGA1. Therefore, targeting the NUDT16-HMGA1 pathway may be a novel strategy to enhance the sensitivity of radiotherapy and chemotherapy.

Complete and accurate DNA replication is essential for cell proliferation and genome stability. However, various factors such as DNA damage and transcription-replication conflict can interrupt or even terminate DNA replication, known as “replication stress” (1). When the replication barrier cannot be treated in a timely manner, replication fork progression will be terminated, resulting in varying degrees of genomic alterations, which can lead to cell death in severe cases (1, 2, 3, 4). Numerous studies have shown that persistent replication pressure exists in tumor cells and represents an important cause of genomic instability (2, 3). Due to the persistent replication pressure in tumor cells, chemotherapy and radiotherapy could aggravate replication pressure to alleviate tumor progression, which has become an important strategy for cancer treatment (5, 6, 7, 8, 9). Nevertheless, the efficacy of current chemotherapy drugs and radiotherapy is limited in some cases, and more efficient therapeutic targets need to be explored.

When cells encounter replication stress, the stability of stalled replication forks is reduced, and the protection of stalled replication forks is essential to cope with replication stress (10, 11, 12). The arrest of replication fork progression results in ssDNA exposure, followed by replication protein A (RPA) binding to ssDNA and stabilizing the replication fork (11, 13). Subsequently, replication protein A can recruit the DNA translocation enzyme SMARCAL1 to mediate replication fork reversal (14). In addition to SMARCAL1, other SNF2 family members, such as ZRANB3, HLTF, as well as topoisomerase IIα (TOP2A), also mediate replication fork reversal (15, 16, 17). Many other proteins are still required to protect replication forks from degradation after inversion, including BRCA2, RAD51, and FANCD2 (18, 19, 20). FANCD2 inhibits nuclease DNA2 activity through its N-terminal domain. In addition, FANCD2 collaborates with RAD51 to inhibit the activity of multiple nucleases, including DNA2, MRE11, and EXO1 (21). Replication fork reversal and inhibition of replication fork degradation are considered key processes to cope with replication stress, which is crucial for the survival and proliferation of tumor cells. Therefore, targeting replication fork stabilization may represent a novel strategy for enhancing sensitivity to chemotherapy and radiotherapy.

High mobility group A1 (HMGA1) is a small nuclear protein, which is bound to chromosomes as a third class of chromosomal proteins (22). HMGA1 is termed a "structural transcription factor" given that it can alter the chromatin structure and promote interaction between transcriptional regulatory proteins and DNA (23). HMGA1 is expressed at low levels in normal tissues but is highly expressed in a variety of malignant tumors, including breast, liver, lung, bladder, and ovarian cancers. Its high expression could significantly promote the proliferation of tumor cells and is closely related to poor prognosis (24, 25, 26). Additionally, numerous studies have shown that HMGA1 is closely correlated with chemotherapy and radiotherapy resistance (27, 28). HMGA1 not only promotes the expression of ataxia-telangiectasia mutated, a key regulator of DNA damage repair, but also acts as its downstream molecule to promote DNA damage repair and enhance the chemotherapy and radiotherapy resistance of tumor cells (28, 29). HMGA1 has also been shown to enhance base excision repair through its dRP/AP site cleavage activities and promote chemoresistance of tumor cells (30). However, whether and how HMGA1 is involved in maintaining replication fork stability remains to be explored.

In this study, we found that the expression of HMGA1 was higher during the S phase, while inhibition of HMGA1 reduced the stability of replication forks and proliferation of tumor cells. We also showed that PARP1 mediates PARylation of HMGA1 at glutamic acid (Glu, E) 50, whereas NUDT16 mediates the dePARylation of HMGA1, preventing the targeted degradation of PARylated HMGA1 by the E3 ubiquitin ligase CHFR. Moreover, inhibition of the NUDT16-HMGA1 pathway significantly enhanced the efficacy of chemotherapy and irradiation (IR) treatment. These results suggest that HMGA1 is involved in the maintenance of replication fork stability and that NUDT16 positively regulates HMGA1 protein expression. The NUDT16-HMGA1 pathway may be a novel strategy for cancer treatment.

## Results

### HMGA1 depletion significantly inhibits DNA replication and cell proliferation

**Figure 1:**
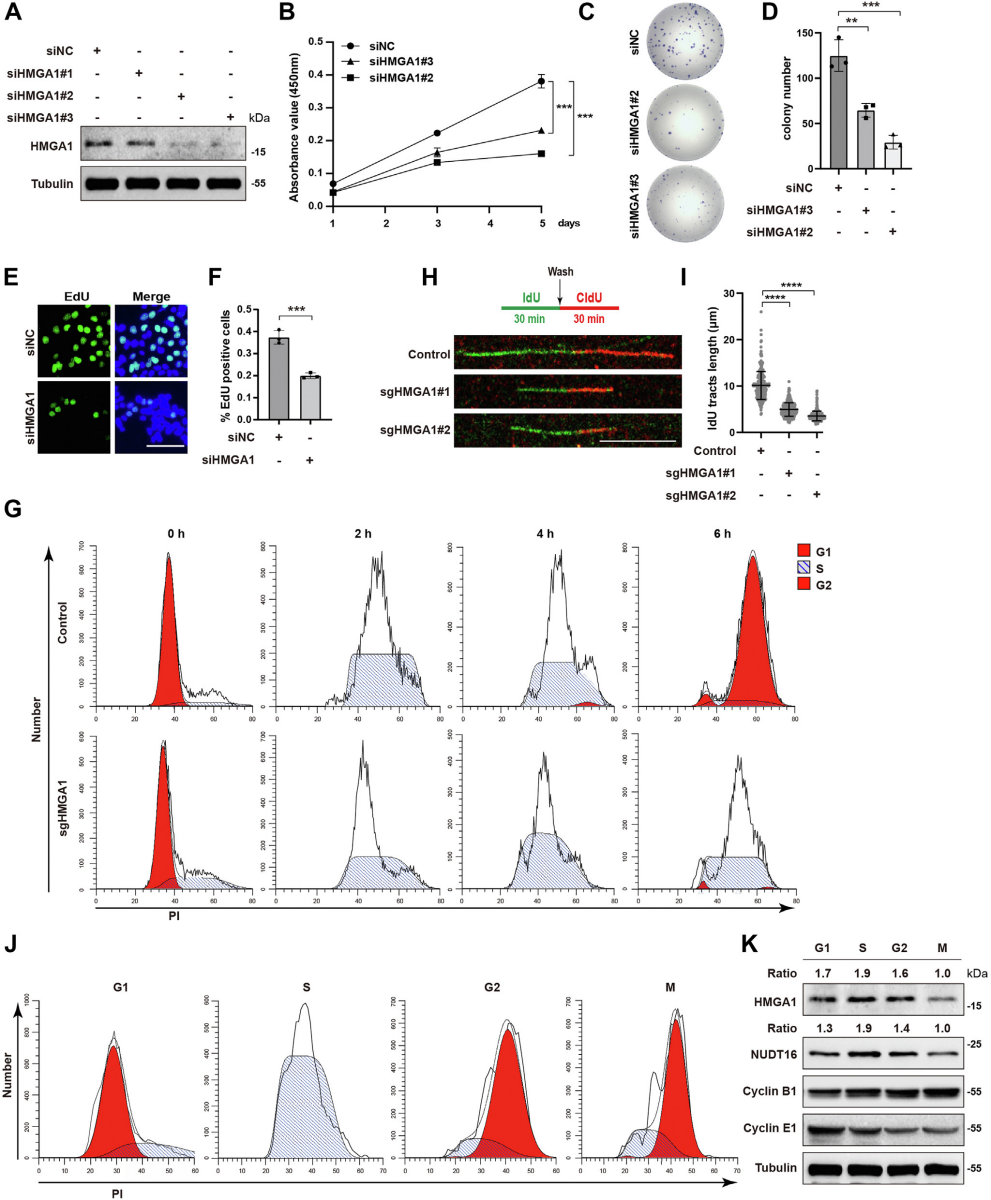
**HMGA1 depletion significantly inhibits DNA replication and cell proliferation.***A*–*D*, HMGA1 depletion significantly inhibited the proliferation of HCC-LM3 cells. HCC-LM3 cells were transfected with HMGA1 siRNAs for 48 h, and the HMGA1 protein levels were examined by western blotting analysis (*A*). After HCC-LM3 cells were transfected with HMGA1 siRNAs, cell proliferation was detected by CCK-8 assay at day 1, day 3, and day 5 (n = 3) (*B*), and by clonal formation assay (n = 3) (*C* and *D*). *E*–*G*, HMGA1 depletion significantly inhibited S phase progression in both HCC-LM3 and HeLa cells. EdU assay was used to detect the proportion of S-phase cells in HCC-LM3 HMGA1-knockdown cells (n = 3). The scale bar represents 100 μm. *E* and *F*, after synchronized treatment of HeLa HMGA1-KO cells and control cells, the duration of S phase was measured by flow cytometry (*G*). *H* and *I*, endogenous HMGA1 deletion severely impaired normal DNA replication. Replication trajectories of DNA were visualized by CldU and IdU, and CldU lengths were measured in both HeLa control cells and HMGA1-KO cells. The scale bar represents 10 μm. At least 300 trajectories were measured for each group. *J* and *K*, the protein level of HMGA1 and NUDT16 was highest in S phase. After the synchronization treatment, the G1, S, G2, and M phases were determined by flow cytometry, and then the cells were collected at the corresponding time points. The protein level was detected by western blotting analysis. CCK-8, Cell Counting Kit-8; HCC, hepatocellular carcinoma; HMGA1, high mobility group A1.

HMGA1 has been reported to act as a structural transcription factor to promote the transcription of oncogenes and tumor progression by regulating autophagy, angiogenesis, and epithelial-mesenchymal transition (22, 23, 26). Public databases show that HMGA1 protein is highly expressed in many cancers, including hepatocellular carcinoma (HCC), lung adenocarcinoma, and pancreatic cancer, and patients with high HMGA1 expression have a worse prognosis than those with low HMGA1 expression (Fig. S1, *A* and *B*). We next verified the effect of HMGA1 on cell proliferation by Cell Counting Kit-8 and colony formation assays. As shown in Figures 1, *A*–*D* and S1, *C*–*F*, HMGA1 depletion significantly inhibited cell proliferation. The results of EdU assay showed that HMGA1 depletion significantly reduced the proportion of S-phase cells (Figs. 1, *E* and *F* and S1, *G* and *H*). Additionally, flow cytometry showed that KO of HMGA1 resulted in a marked prolongation of the S phase (Fig. 1*G*). These results suggest that HMGA1 promotes S phase progression. Given that complete and accurate DNA replication is essential for S phase progression, we further explored whether HMGA1 affects DNA replication through a DNA fiber assay. As shown in Figure 1, *H* and *I*, HMGA1 depletion significantly inhibited nascent DNA strand elongation, indicating that HMGA1 is required for DNA replication. Notably, the protein expression of HMGA1 was highest during the S phase (Fig. 1, *J* and *K*). These results suggest that HMGA1 plays a pivotal role in DNA replication and cell proliferation.

### HMGA1 promotes DNA replication by protecting replication forks

**Figure 2:**
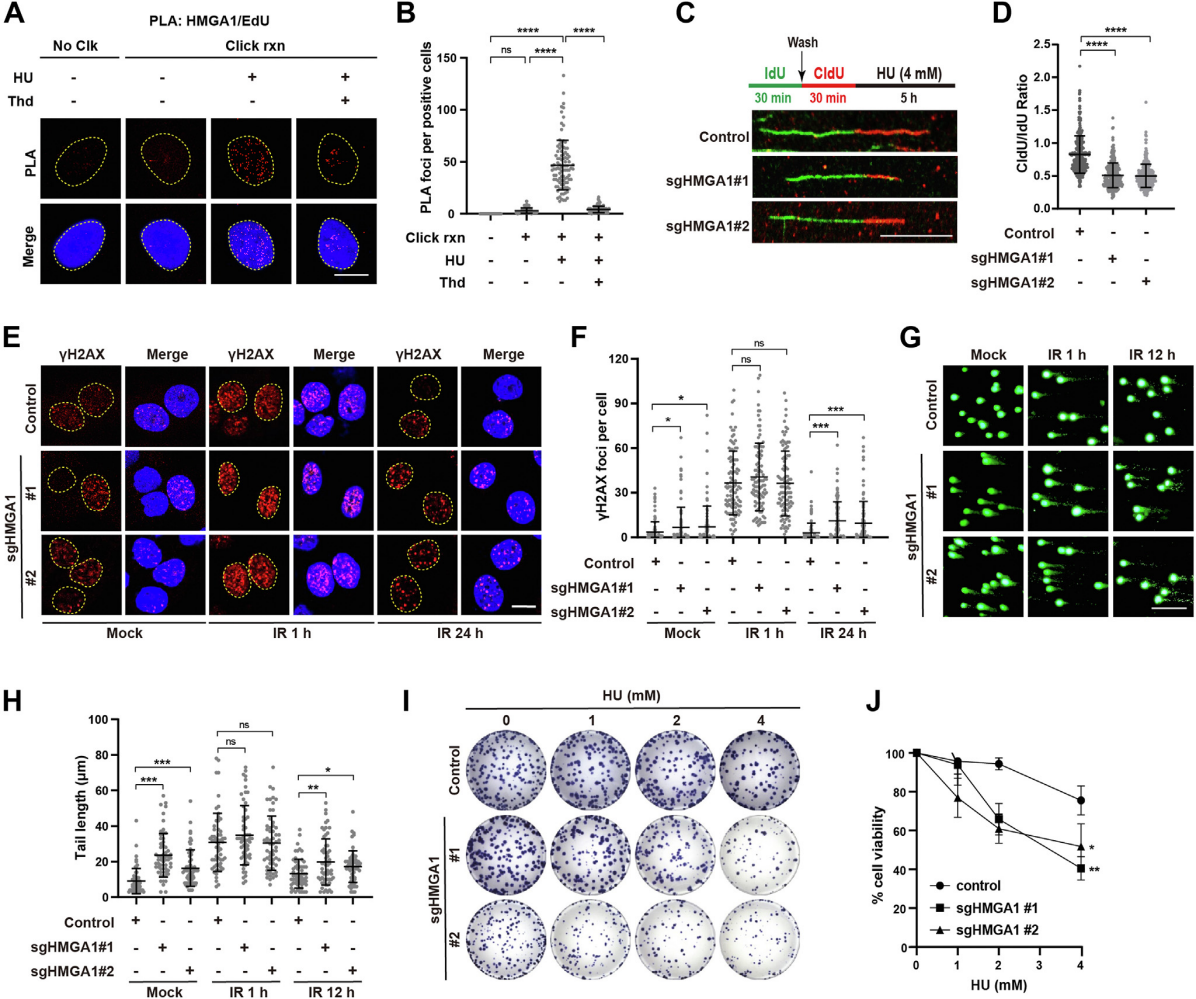
**HMGA1 promotes DNA replication by protecting replication forks.***A* and *B*, analysis of HMGA1 recruitment by PLA. HeLa cells were pulse-labeled with 10 μM EdU for 15 min followed with or without 10 μM thymidine chase for 1 h (Thd). Cells were then left untreated or treated with 4 mM HU for 3 h and subjected to PLA with anti-HMGA1 and anti-biotin antibodies. The scale bar represents 10 μm. Quantification of HMGA1/biotin-EdU PLA foci number per focus positive cell. At least 100 cells were counted in each individual experiment. *C* and *D*, depletion of endogenous HMGA1 markedly accelerated the degradation of stalled replication forks. CldU and IdU were used to visualize the replication trajectories of DNA, and the CldU and IdU lengths were measured in Control and HMGA1-KO HeLa cells to calculate the ratio of CldU: IdU. At least 300 trajectories were measured for each group. The scale bar represents 10 μm. *E* and *F*, depletion of HMGA1 resulted in more γ-H2AX foci and prolonged presence of γ-H2AX foci. Control or HMGA1-KO HeLa cells were either untreated or treated with IR (4Gy), and then the γ-H2AX foci at different time points was visualized by immunofluorescence. γ-H2AX foci were quantified using ImageJ software. At least 100 cells were analyzed for each group. The scale bar represents 10 μm. *G* and *H*, depletion of HMGA1 resulted in more DNA damage and inhibition of DNA repair. Control or HMGA1-KO HeLa cells were either untreated or treated with IR (4Gy), and then the comet tail at different time points was detected by alkaline comet assay. CASP software was used to quantify the length of the comet tail to determine the level of DNA breakage. At least 60 comet tails were analyzed for each group. The scale bar represents 100 μm. *I* and *J*, depletion of HMGA1 markedly enhanced the sensitivity of HeLa cells to HU treatment. Control and HMGA1-KO HeLa cells were treated with various concentrations of HU for 24 h, and were continued in culture to form colonies. Colonies were quantified using ImageJ software. HMGA1, high mobility group A1; HU, hydroxyurea; IR, irradiation; PLA, proximity ligation assay.

DNA replication often encounters various obstacles leading to replication fork arrest, which is called "replication stress", especially in tumor cells (2). The stability of nascent DNA strands at stalled replication forks is greatly reduced, and protecting stalled replication forks is essential to restart the replication process (31, 32, 33). To clarify whether HMGA1 is involved in protecting stalled replication forks, we first treated cells with hydroxyurea (HU), a nucleotide reductase inhibitor that specifically inhibits DNA synthesis, to induce replication stress. Then, the *in situ* proximity ligation assay (PLA) was used to determine whether HMGA1 localizes to the stalled replication forks. As shown in Figure 2, *A* and *B*, HMGA1 was significantly enriched at the stalled replication forks after HU treatment. Furthermore, the results of the DNA fiber assay showed that HMGA1 KO aggravated the degradation of nascent DNA strands at the stalled replication forks after treatment with HU (Fig. 2, *C* and *D*). These results reveal that when cells encounter replication stress, HMGA1 is recruited to the stalled replication forks and subsequently protects them. Moreover, HMGA1 depletion accelerates stalled replication fork degradation, resulting in more severe DNA damage. As shown in Figure 2, *E*–*H*, HMGA1 KO resulted in longer comet tails and more γH2AX foci, with a significantly slower recovery rate of comet tails and γH2AX foci after IR. Finally, we employed colony formation assays to examine the effect of HMGA1 depletion on the ability of cells to cope with replication stress. As shown in Figure 2, *I* and *J*, KO of HMGA1 significantly reduced cell tolerance of replication stress. These results indicate that HMGA1 promotes DNA replication by protecting replication forks.

### HMGA1 maintains replication fork stability by recruiting FANCD2

**Figure 3:**
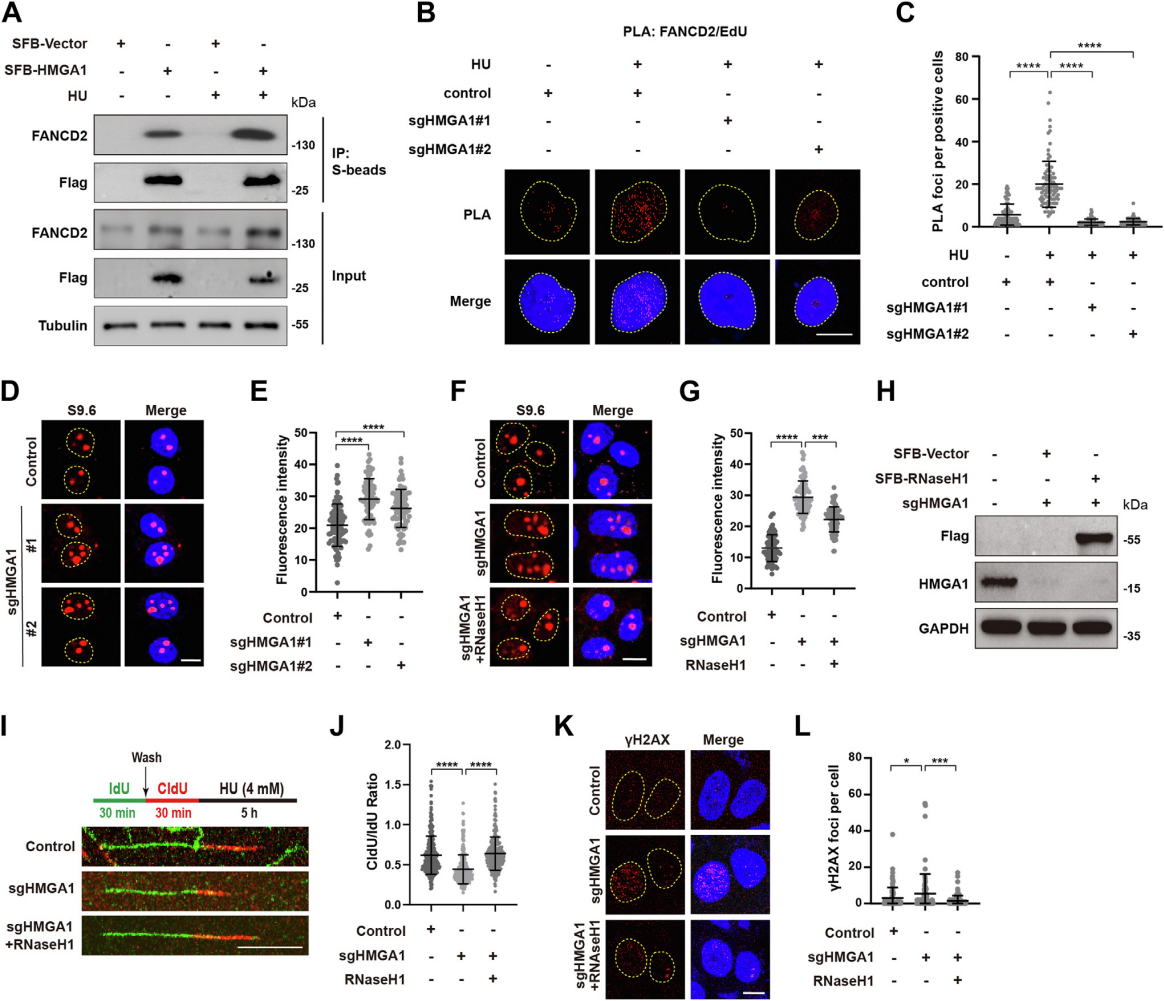
**HMGA1 maintains replication fork stability by recruiting FANCD2.***A*, HMGA1 interacted with FANCD2 after HU treatment. HeLa cells were transiently transfected with the indicated plasmids for 24 h and treated with 4 mM HU for 3 h, then the cells were collected and lysed for co-IP with anti-S beads and analyzed by western blotting with indicated antibodies. *B* and *C*, HMGA1 KO significantly reduces FANCD2/biotin-EdU PLA foci formation. Control or HMGA1-KO HeLa cells were pulse-labeled with 10 μM EdU for 15 min followed with or without 4 mM HU for 3 h and subjected to PLA with anti-FANCD2 and anti-biotin antibodies. The scale bar represents 10 μm. Quantification of FANCD2/biotin-EdU PLA foci number per focus positive cell. At least 100 cells were counted in each individual experiment. *D*–*H*, depletion of HMGA1 significantly enhanced S9.6 signaling in the nucleus. S9.6 signaling of control or HMGA1-KO HeLa cells was visualized by immunofluorescence. The scale bar represents 10 μm. The fluorescence intensity of S9.6 was quantified using ImageJ software. At least 100 cells were analyzed for each group (*D* and *E*). S9.6 signaling of control and HMGA1-KO HeLa cells transfected with empty vector or SFB-RNaseH1 was visualized by immunofluorescence. The scale bar represents 10 μm. The fluorescence intensity of S9.6 was quantified using ImageJ software. At least 100 cells were analyzed for each group (*F*–*H*). *I* and *J*, accumulation of S9.6 markedly accelerated the degradation of stalled replication forks. CldU and IdU were used to visualize the replication trajectories of DNA, and the CldU and IdU lengths were measured in control, HMGA1-KO, or HMGA1-KO + SFB-RNaseH1 HeLa cells to calculate the ratio of CldU: IdU. The scale bar represents 10 μm. At least 300 trajectories were measured for each group. *K* and *L*, the enhanced S9.6 signal induced by depletion of HMGA1 significantly increased γH2AX foci. γH2AX foci of control or HMGA1-KO or HMGA1-KO + SFB-RNaseH1 HeLa cells was visualized by immunofluorescence. The scale bar represents 10 μm. The γH2AX foci were quantified using ImageJ software. At least 100 cells were analyzed for each group. co-IP, co-immunoprecipitation; HMGA1, high mobility group A1; HU, hydroxyurea; PLA, proximity ligation assay.

To elucidate the mechanism by which HMGA1 maintains replication fork stability, the interactors of HMGA1 were reviewed in the BioGRID database, and several proteins reported to be involved in replication fork stability were validated by co-immunoprecipitation (co-IP) assay. As shown in Fig. S1*J*, HMGA1 interacted with FANCD2, FANCI, and RAD51, especially with FANCD2. In addition, HU treatment clearly enhanced the interaction between HMGA1 and FANCD2 (Fig. 3*A*). FANCD2 has been reported to be recruited to stalled replication forks, protecting them from degradation by inhibiting the activity of multiple nucleases (21, 34, 35). To determine whether the interaction of HMGA1 with FANCD2 affects the enrichment of FANCD2 at stalled replication forks, we used the PLA to measure FANCD2/biotin-EdU PLA focus formation after deletion of HMGA1. As shown in Figure 3, *B* and *C*, HMGA1 KO significantly reduced the number of FANCD2/biotin-EdU PLA foci, suggesting that HMGA1 is essential for FANCD2 localization to the stalled replication fork.

In addition to protecting stalled replication forks, FANCD2 is also involved in downregulating the R-loops (36). The R-loop is a three-stranded structure of RNA–DNA hybrids, which is one of the important sources of replication stress. To further explore whether HMGA1 affected the accumulation of R-loops, we used S9.6 antibodies to measure the levels of R-loops. As shown in Figure 3, *D* and *E*, KO of HMGA1 greatly increased the proportion of R-loops in the nucleus. Overexpression of RNaseH1, an RNA endonuclease that specifically degrades R-loops, reversed the effects of HMGA1 depletion on the accumulation of R-loop (Fig. 3, *F*–*H*). These results suggest that HMGA1 is involved in resolving R-loops. Additionally, aberrant accumulation of R-loop leads to stalled replication forks and DNA damage (37, 38). Overexpression of RNaseH1 reduced the degradation of nascent DNA strands and the number of γH2AX foci caused by HMGA1 depletion (Fig. 3, *I*–*L*). These results suggest that HMGA1 plays a crucial role in reducing the degradation of nascent DNA strands and DNA damage at the stalled replication forks induced by R-loops.

### NUDT16 mediates HMGA1 dePARylation and enhances its protein stability

**Figure 4:**
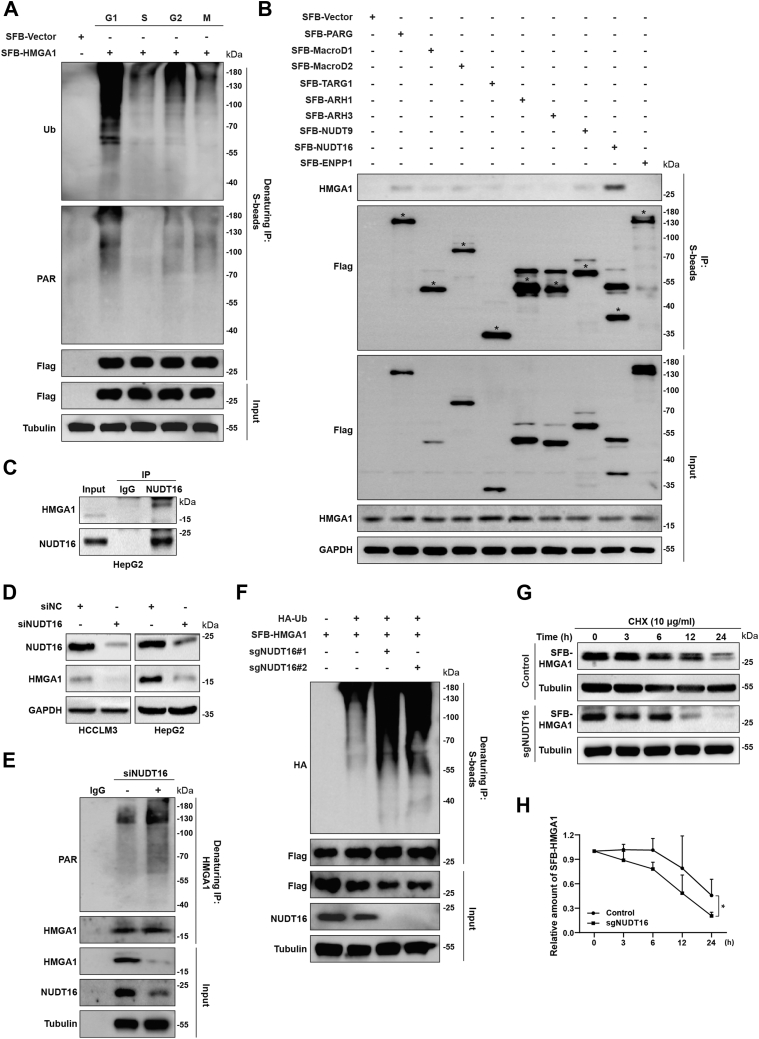
**NUDT16 mediates HMGA1 dePARylation and enhances its protein stability.***A*, after transient transfection with SFB-Vector and SFB-HMGA1, HeLa cells overexpressing SFB-HMGA1 were synchronized and then released. Cells in G1, S, M, and G2 phases were collected and lysed for denaturing co-IP assay with anti-S beads, followed by western blotting with indicated antibodies. *B* and *C*, NUDT16 interacted with HMGA1. HEK293T cells were transiently transfected with the indicated plasmids for 24 h, and the cells were collected and lysed for co-IP with anti-S beads and analyzed by western blotting with indicated antibodies (*B*). HepG2 cells were collected and lysed for co-IP with anti-IgG or anti-NUDT16 antibodies and analyzed by western blotting with the indicated antibodies (*C*). *D*–*H*, NUDT16 enhanced HMGA1 protein stability by reversing its PARylation. After transfection of HepG2 and HCC-LM3 cells with NUDT16 siRNA for 48 h, the cells were collected and lysed for western blotting with the indicated antibodies (*D*). HeLa cells were transfected with NUDT16 siRNA for 48 h, and cells were collected and lysed for denaturing co-IP with anti-IgG or anti-HMGA1 antibodies and analyzed by western blotting with the indicated antibodies (*E*). Control and NUDT16-KO HeLa cells were transiently transfected with SFB-HMGA1 and HA-Ub for 24 h, followed by incubation with MG132 (10 μM) for another 6 h. And then the cells were collected and lysed for denaturing co-IP with anti-S beads and analyzed by western blotting with the indicated antibodies (*F*). *G* and *H*, control and NUDT16-KO HeLa cells transfected with SFB-HMGA1 were treated with 10 μg/ml cycloheximide (CHX) for 0, 3, 6, 12, and 24 h, respectively. Cells were collected and lysed for western blotting. HMGA1 protein levels were quantified using ImageJ software (n = 3). co-IP, co-immunoprecipitation; HCC, hepatocellular carcinoma; HMGA1, high mobility group A1; IgG, immunoglobulin G.

Given the role of HMGA1 in DNA replication and cell proliferation, we further explored the regulatory mechanism of HMGA1. HMGA1 has the highest protein expression level during S phase (Fig. 1, *J* and *K*); however, we found no significant difference in HMGA1 mRNA in each stage of the cell cycle (Fig. S1*I*). Therefore, we hypothesized that the differential expression of HMGA1 in different phases might be related to protein posttranslational modification, among which, PARylation and ubiquitination are the main types of modifications that regulate protein degradation (39, 40). As shown in Figures 4*A* and S2, *A* and *B*, HMGA1 indeed modified with PAR, and had the lowest level of PARylation and ubiquitination modification during the S phase. Next, we screened dePARylation enzymes by co-IP assay, and found that NUDT16 had the most pronounced interaction with HMGA1, suggesting that NUDT16 most likely mediates the dePARylation of HMGA1 (Figs. 4, *B* and *C* and S2*C*). Therefore, we investigated the effect of NUDT16 depletion on HMGA1 protein and mRNA levels. As shown in Figures 4*D* and S2, *D* and *E*, NUDT16 depletion dramatically reduced HMGA1 protein expression levels but not mRNA levels. To note, the protein levels of NUDT16 and HMGA1 were synchronously altered throughout the cell cycle, which suggested that the regulation of HMGA1 expression levels by NUDT16 is unaffected by the cell cycle (Fig. S2*F*). Furthermore, NUDT16 depletion also significantly increased the levels of PARylation of HMGA1 (Figs. 4*E* and S2, *G* and *H*).

Given that many PARylated proteins are subsequently recognized by E3 ubiquitin ligases to mediate ubiquitination (41), we further explored whether NUDT16 affected HMGA1 ubiquitination level through dePARylation and ultimately regulated HMGA1 protein expression. As shown in Figure 4*F*, depletion of NUDT16 greatly increased HMGA1 ubiquitination. It has been reported that ubiquitination includes various branched ubiquitin chains; for example, K11/K48-linked polyubiquitylation mediates proteasomal degradation of substrate proteins, while K63-linked polyubiquitylation mediates functional alteration of substrate proteins (42). Ubiquitin mutants were constructed to clarify which ubiquitin branched link of HMGA1 is specifically affected by NUDT16. As shown in Fig. S2*I*, deletion of NUDT16 markedly increased K48-linked polyubiquitylation but not other chain-linked polyubiquitylation (K6, K11, K27, K29, K33, and K63). Consistently, the deletion of NUDT16 also markedly shortened the protein half-life of HMGA1 (Fig. 4, *G* and *H*). These results suggest that NUDT16 reduced the K48-linked polyubiquitylation of HMGA1 by reversing its PARylation, thereby enhancing its protein stability.

### PARP1 mediates PARylation of HMGA1 at E50

**Figure 5:**
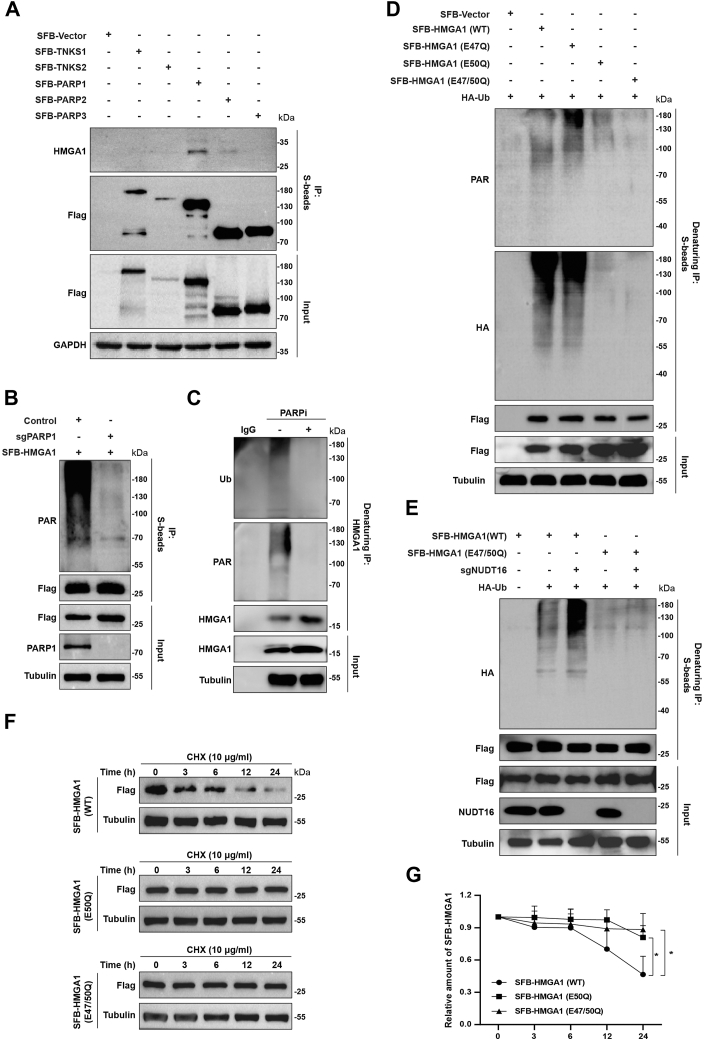
**PARP1 mediates the PARylation of HMGA1 at E50.***A*, PARP1 interacts with HMGA1. HEK293T cells were transiently transfected with the indicated plasmids for 24 h, and the cells were collected and lysed for co-IP with anti-S beads and analyzed by western blotting with indicated antibodies. *B*, PARP1 mediates the PARylation of HMGA1. Control and PARP1-KO HeLa cells were transiently transfected with SFB-HMGA1 for 24 h, and then the cells were collected and lysed for co-IP with anti-S beads and analyzed by western blotting with the indicated antibodies. *C*, HeLa cells were treated with 10 μM BMN673 for 24 h, and the cells were collected and lysed for denaturing co-IP with anti-IgG or anti-HMGA1 antibodies and analyzed by western blotting with the indicated antibodies. *D*, HEK293T cells were transiently transfected with SFB-HMGA1 (WT), SFB-HMGA1 (E47Q), SFB-HMGA1 (E50Q) or SFB-HMGA1 (E47/50Q) for 24 h, followed by incubation with MG132 (10 μM) for another 6 h. And the cells were collected and lysed for denaturing co-IP with anti-S beads and analyzed by western blotting with indicated antibodies. *E*, control and NUDT16-KO HeLa cells were transiently transfected with SFB-HMGA1 (WT), or SFB-HMGA1 (E47/50Q) and HA-Ub for 24 h, followed by incubation with MG132 (10 μM) for another 6 h. And then the cells were collected and lysed for denaturing co-IP with anti-S beads and analyzed by western blotting with the indicated antibodies. *F* and *G*, mutations in Glu 50 prolong the protein half-life of HMGA1. HeLa cells were transiently transfected with SFB-HMGA1 (WT), SFB-HMGA1 (E50Q), or SFB-HMGA1 (E47/50Q) were treated with 10 μg/ml cycloheximide (CHX) for 0, 3, 6, 12, and 24 h, respectively. Cells were collected and lysed for western blotting. HMGA1 protein levels were quantified using ImageJ software (n = 3). co-IP, co-immunoprecipitation; HMGA1, high mobility group A1; IgG, immunoglobulin G.

We next conducted co-IP assay to identify the enzymes that mediate HMGA1 PARylation. Figure 5, *A* and *B* show that the interaction between PARP1 and HMGA1 was most pronounced, and PARP1 KO markedly abolished the PARylation of HMGA1, suggesting that PARP1 is the most dominant enzyme mediating HMGA1 PARylation. Further, we evaluated the effect of PARP inhibitors on HMGA1 PARylation and ubiquitination. As shown in Figure 5*C*, PARP inhibitor BMN673 treatment significantly inhibited the PARylation and ubiquitination of HMGA1. To map the PARylation site in HMGA1, we mutated the E47, E50, and E47/50 of SFB-HMGA1 based on the data from previous studies (43). As shown in Figure 5*D*, the E50 and E47/50 mutants, but not the E47 mutant, significantly downregulated HMGA1 PARylation and ubiquitination. Moreover, NUDT16 depletion increased the ubiquitination of HMGA1 (WT) but not HMGA1 (E47/50Q mutant) (Fig. 5*E*). Given that E50 is the predominant site of HMGA1 PARylation, we proceeded to test whether mutating E50 impacted the protein half-life of HMGA1. As shown in Figure 5, *F* and *G*, the protein half-life of HMGA1 (E50Q and E47/50Q mutants) was significantly longer than that of HMGA1 (WT). The above results indicate that PARP1 mediates the PARylation of HMGA1 at E50.

### E3 ubiquitin ligase CHFR mediates ubiquitination of PARylated HMGA1

**Figure 6:**
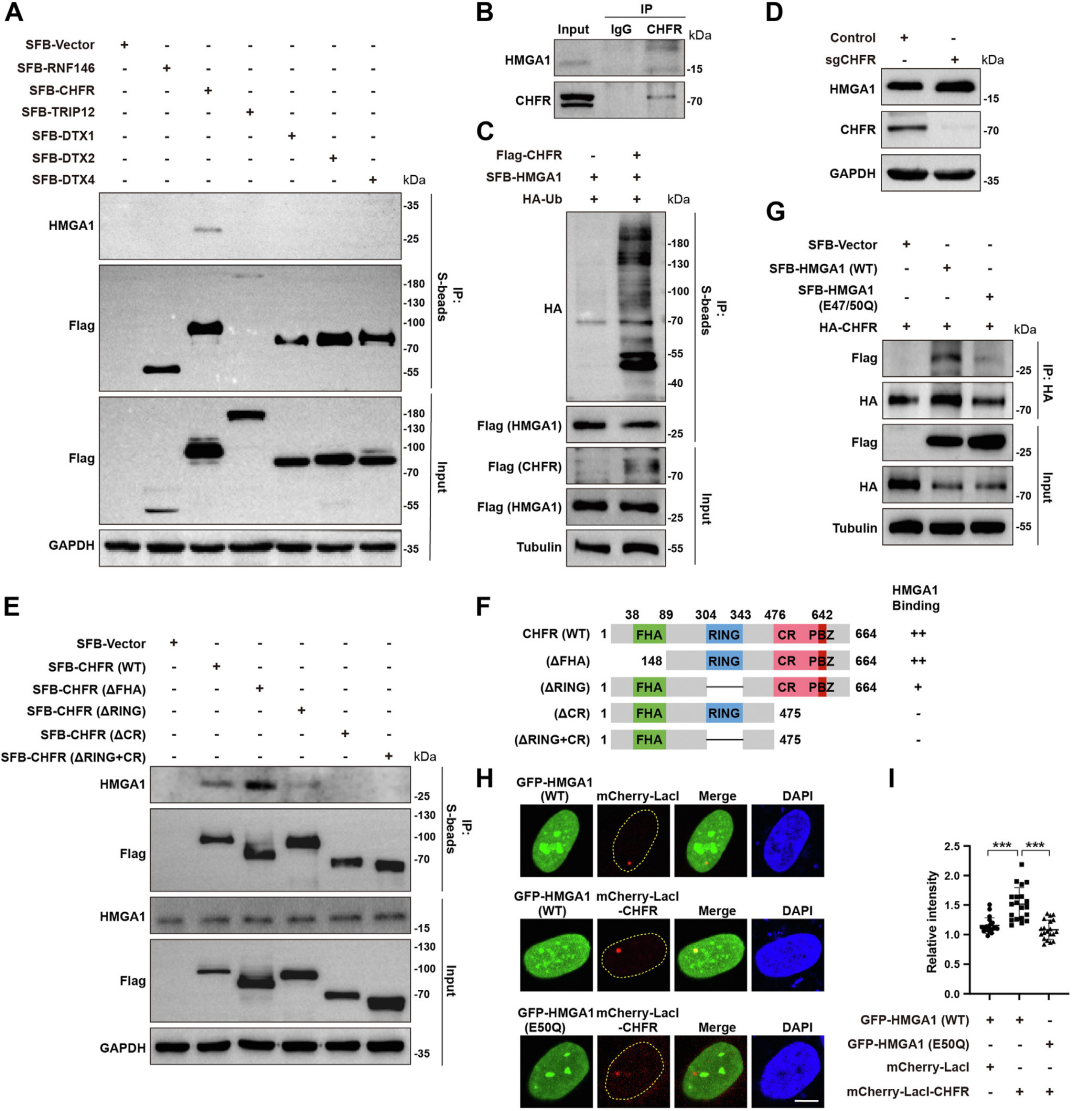
**The E3 ubiquitin ligase CHFR mediates ubiquitination of PARylated HMGA1.***A* and *B*, CHFR interacts with HMGA1. HEK293T cells were transiently transfected with indicated plasmids for 24 h, and the cells were collected and lysed for co-IP with anti-S beads and analyzed by western blotting with indicated antibodies (*A*). HeLa cells were collected and lysed for co-IP with anti-IgG or anti-CHFR antibodies and analyzed by western blotting with the indicated antibodies (*B*). *C* and *D*, CHFR mediates HMGA1 ubiquitination and downregulates its protein level. HEK293T cells were transiently transfected with SFB-HMGA1, Flag-CHFR and HA-Ub for 24 h, followed by incubation with MG132 (10 μM) for another 6 h. And the cells were collected and lysed for Co-IP with anti-S beads and analyzed by western blotting with indicated antibodies (*C*). Control and CHFR-KO HeLa cells were collected and lysed for western blotting with the indicated antibodies (*D*). *E* and *F*, CHFR recognizes HMGA1 through the PBZ domain. The diagram of CHFR domains is shown in (*F*). HEK293T cells were transiently transfected with SFB-CHFR (WT) or its deletion mutants, and the cells were collected and lysed for co-IP with anti-S beads and analyzed by western blotting with indicated antibodies (*E*). *G*–*I*, HMGA1 recognition by CHFR is dependent on PARylation at the Glu 50 of HMGA1. HEK293T cells were transiently transfected with HA-CHFR, SFB-HMGA1 (WT), or SFB-HMGA1 (E47/50Q), and the cells were collected and lysed for co-IP with anti-HA beads and analyzed by western blotting with indicated antibodies (*G*). U2OS cells with stable integration of LacO arrays (U2OS-265) were cotransfected with GFP-HMGA1 and mCherry-LacI-CHFR for 24 h. HMGA1 and CHFR were visualized by immunofluorescence, and the fluorescence intensity of GFP-HMGA1 foci was quantified using ImageJ software (*H* and *I*). co-IP, co-immunoprecipitation; HMGA1, high mobility group A1; IgG, immunoglobulin G; PBZ, PAR-binding zinc finger.

When substrate proteins undergo PARylation, they are recognized by E3 ubiquitin ligases, which mediate their ubiquitination (44). We next investigated the E3 ubiquitin ligases that mediate the ubiquitination of PARylated HMGA1. The interaction between CHFR and HMGA1 was identified by endogenous and exogenous co-IP assays, which initially suggested that the E3 ligase CHFR mediates HMGA1 ubiquitination. For further validation, we overexpressed Flag-CHFR and found that it significantly increased the ubiquitination of HMGA1 (Fig. 6*C*). Additionally, KO of CHFR markedly upregulated HMGA1 expression (Fig. 6*D*). Therefore, we clarified that CHFR mediates HMGA1 ubiquitination. To understand how CHFR interacts with HMGA1, we mapped the binding domains between CHFR and HMGA1 by co-IP assay. As shown in Figure 6, *E* and *F*, SFB-CHFR (WT) and SFB-CHFR (▵FHA) showed significant interaction with HMGA1, while SFB-CHFR (▵RING) showed weak interaction with HMGA1. However, SFB-CHFR (▵CR) and SFB-CHFR (▵RING + CR) did not interact with HMGA1. These results indicate that HMGA1 binds to the PAR-binding zinc finger (PBZ) domain of CHFR, and that the RING domain may be essential for the binding of HMGA1 and CHFR. To determine whether the binding of HMGA1 to CHFR is dependent on PARylation, we performed co-IP and LacI-LacO fluorescence colocalization assays. As shown in Figure 6*G*, the interaction between CHFR and HMGA1 (E47/50Q) was greatly attenuated compared to HMGA1 (WT). Consistent with this result, LacI-LacO fluorescence colocalization assays showed significantly stronger colocalization of mCherry-lacI-CHFR with GFP-HMGA1 (WT) than with GFP-HMGA1 (E47/50Q) (Fig. 6, *H* and *I*). These results indicate that CHFR mediates the ubiquitination of PARylated HMGA1.

### Inhibition of NUDT16-HMGA1 pathway increases DNA damage and enhances the efficacy of chemotherapy and IR treatment

**Figure 7:**
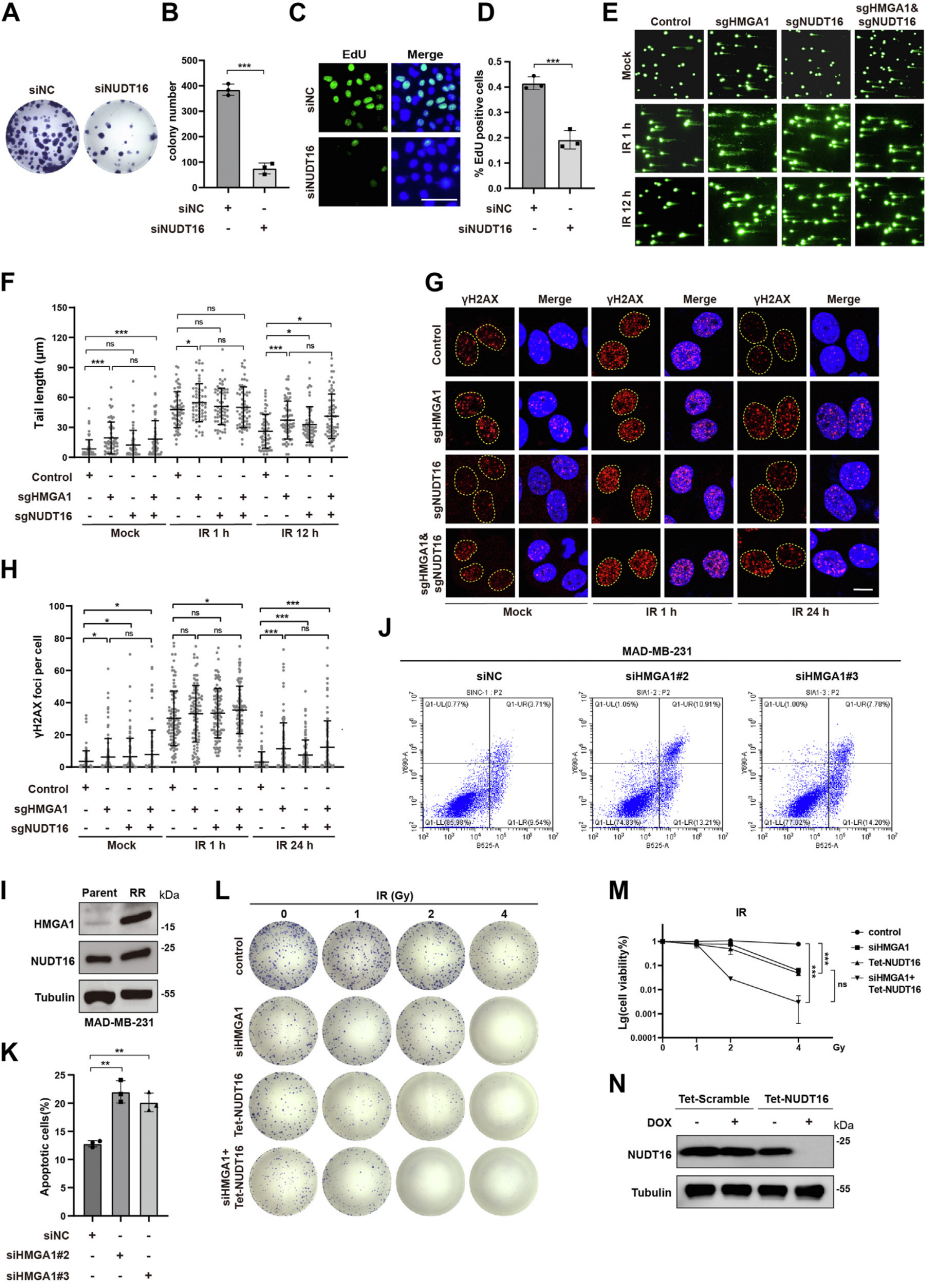
**Inhibition of NUDT16-HMGA1 pathway increases DNA damage and enhances the efficacy of chemotherapy and IR treatment.***A*–*D*, NUDT16 depletion inhibits cell proliferation. After HCC-LM3 cells were transfected with NUDT16 siRNAs, cell proliferation was detected by clonal formation assay (n = 3) (*A* and *B*). EdU assay was used to detect the proportion of S phase cells in HCC-LM3 NUDT16-knockdown cells. The scale bar represents 10 μm. (n = 3) (*C* and *D*). *E*–*H*, inhibition of NUDT16-HMGA1 pathway increases DNA damage. Control, HMGA1-KO, NUDT16-KO, and NUDT16-HMGA1 double KO HeLa cells were either untreated or treated with IR (4Gy), and then the comet tail at different time points was detected by alkaline comet assay. The scale bar represents 10 μm. CASP software was used to quantify the length of the comet tail to determine the level of DNA breakage. At least 60 comet tails were analyzed for each group (*E* and *F*). Control, HMGA1-KO, NUDT16-KO, and NUDT16-HMGA1 double KO HeLa cells were either untreated or treated with IR (4Gy), and then the γ-H2AX foci at different time points was visualized by immunofluorescence. The scale bar represents 10 μm. γ-H2AX foci were quantified using ImageJ software. At least 100 cells were analyzed for each group (*G* and *H*). *I*, the radioresistant breast cancer cell line MAD-MB-231 (231RR) highly expressed HMGA1 and NUDT16. Parental and 231RR cells were collected and lysed, and the protein expression of HMGA1 and NUDT16 were detected by western blotting. *J* and *K*, depletion of HMGA1 increased the proportion of 231RR cells undergoing apoptosis after IR treatment. 231RR cells were transfected with HMGA1 siRNA for 48 h followed by IR (8Gy) treatment or not. The proportion of apoptotic cells was determined by Annexin V/PI staining and flow cytometry after 48 h. *L*–*N*, inhibition of NUDT16-HMGA1 pathway enhances the efficacy of IR treatment. HCC-LM3 cell line stably expressing NUDT16 shRNA (Tet-shNUDT16) was generated. And the cells were transfected with HMGA1 siRNA. Control, NUDT16-depleted, HMGA1-depleted, and both HMGA1 and NUDT16-depleted HCC-LM3 cells were treated with different doses of IR, and were continued in culture to form colonies. Colonies were quantified using ImageJ software. HCC, hepatocellular carcinoma; HMGA1, high mobility group A1; IR, irradiation.

As NUDT16 enhances HMGA1 protein stability through dePARylation and given that HMGA1 promotes cell cycle S phase progression by protecting stalled replication forks from degradation and unwinding R-loops, we next investigated the effect of NUDT16 on S phase progression and cell proliferation. The results of colony formation assays showed that NUDT16 depletion also significantly inhibited cell proliferation (Figs. 7, *A* and *B* and S3, *A*–*D*). Consistently, the results of EdU assay showed that NUDT16 depletion significantly reduced the proportion of S-phase cells (Figs. 7, *C* and *D* and S3, *E* and *F*). Previous studies have shown that NUDT16 promotes DNA damage repair and protects genome stability (45, 46). Therefore, we further explored the effect of HMGA1 and NUDT16 KO on DNA damage. As shown in Figures 7, *E*–*H* and S3*G*, KO of HMGA1 and NUDT16, and NUDT16-HMGA1 double KO all produced longer comet tails and more γH2AX foci, and slowed down the repair process. However, NUDT16-HMGA1 double KO did not significantly aggravate DNA damage and inhibit DNA damage repair compared to HMGA1 KO alone. These results suggest that NUDT16 may promote cell proliferation and DNA stability in part by enhancing HMGA1 protein stability.

Enhancement of DNA damage repair in tumor cells is an important cause of resistance to chemotherapy and radiotherapy (47). Therefore, we further explored whether NUDT16-HMGA1 is associated with chemotherapy and radiotherapy resistance. To this end, we first constructed the IR-resistant MAD-MB-231 cell line (231RR), which was verified by flow cytometry (Fig. S3, *H* and *I*). We noted a marked increase in HMGA1 expression in 231RR compared with parental 231 cells, as well as an increase in NUDT16 expression (Fig. 7*I*). Moreover, compared to the control group, the proportion of 231RR cells undergoing apoptosis after IR treatment was significantly increased in the HMGA1 knockdown group, which suggested that HMGA1 is involved in the resistance of cancer cells to IR exposure (Figs. 7, *J* and *K* and S3*J*). Next, we conducted a colony formation assay with HMGA1 siRNA and Tet-shNUDT16 to investigate the role of the NUDT16-HMGA1 pathway in IR treatment. As shown in Figures 7, *L*–*N* and S3, *L* and *M*, HMGA1 or NUDT16 knockdown alone, and HMGA1-NUDT16 double knockdown all enhanced the sensitivity of HCC-LM3 cells to cisplatin and IR treatment compared to the control group. Notably, compared to HMGA1 or NUDT16 knockdown alone, NUDT16-HMGA1 double knockdown did not enhance the sensitivity of HCC-LM3 cells to cisplatin and IR treatment. The above results suggest that the NUDT16-HMGA1 pathway is closely related to promoting the resistance to chemotherapy and IR treatment, and that NUDT16 enhances the resistance to chemotherapy and IR treatment mostly by enhancing the stability of the HMGA1 protein.

**Figure 8:**
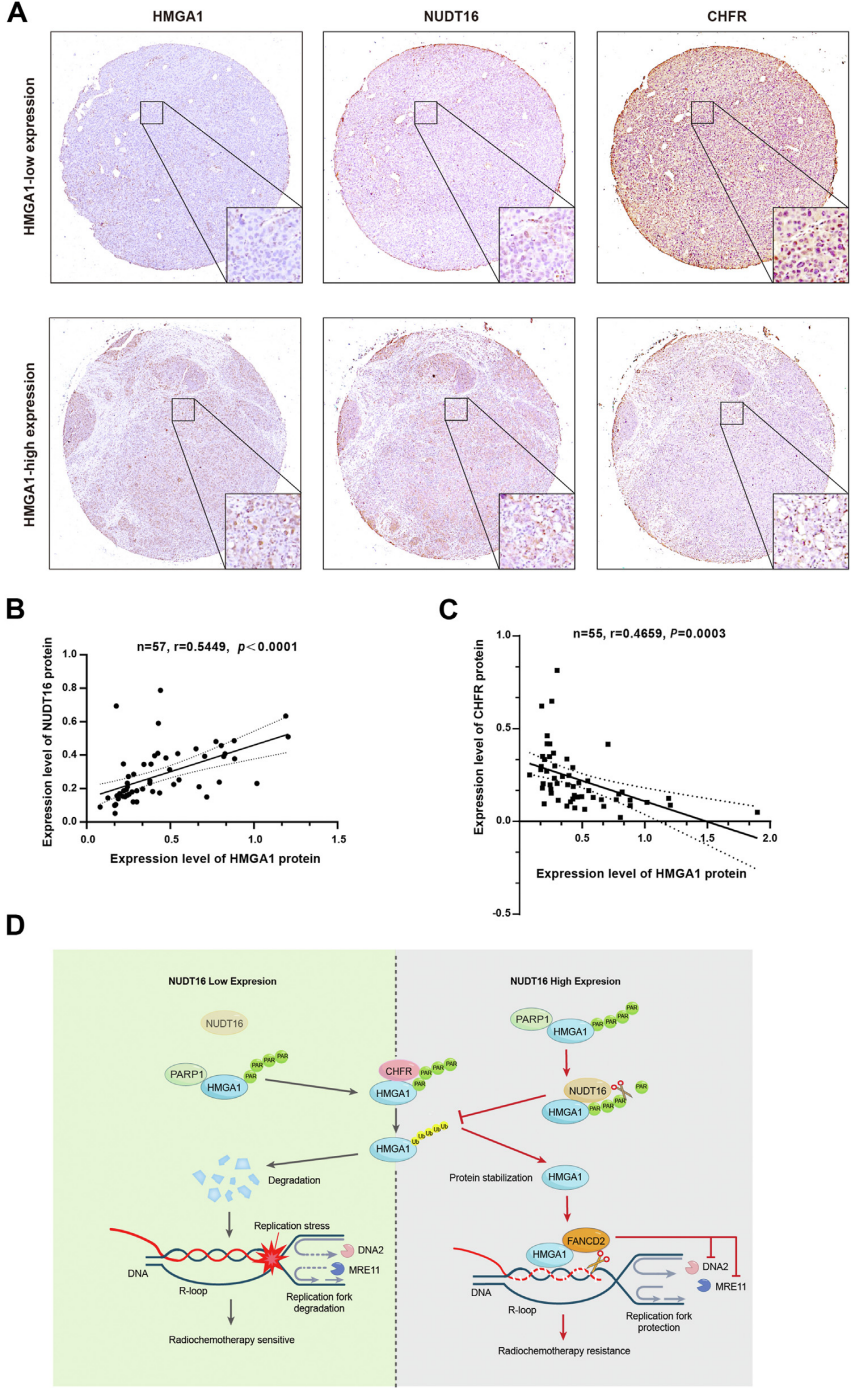
**Inhibition of NUDT16-HMGA1 pathway enhances the efficacy of radiotherapy and chemotherapy.***A*, immunohistochemistry was used to detect the expression level of HMGA1, NUDT16, and CHFR protein in HCC specimens. *B*, the correlation between HMGA1 and NUDT16 protein expression in HCC specimens was analyzed. *C*, the correlation between HMGA1 and CHFR protein expression in HCC specimens was analyzed. *D*, schematic model of the role of NUDT16-HMGA1 pathway in regulating the tolerance of radiotherapy and chemotherapy. HMGA1 recruits FANCD2 to protect stalled replication fork from degradation and reduce R-loop, significantly promoting cell proliferation and resistance to radiotherapy and chemotherapy. PARP1 mediates the PARylation of HMGA1 and promotes the targeted degradation of HMGA1 by E3 ubiquitin ligase CHFR, thereby downregulating its protein expression level. However, dePARylase NUDT16 reverses the PARylation of HMGA1, preventing its subsequent ubiquitin-proteasome degradation pathway and upregulating its protein expression level. HCC, hepatocellular carcinoma; HMGA1, high mobility group A1.

To investigate the potential clinical value of NUDT16-HMGA1 pathway, immunohistochemistry was used to detect the expression of HMGA1, NUDT16, and CHFR in HCC patient specimens. As shown in Figure 8, *A*–*C*, HMGA1 was positively correlated with NUDT16 protein expression and negatively correlated with CHFR protein expression in HCC specimens. In addition, we found that with the increase of pathological grade of HCC specimens, the expression of HMGA1 and NUDT16 increased similarly, while the expression of CHFR decreased (Fig. S4, *A*–*C*). These results are consistent with previous cell experiments. Therefore, targeting the NUDT16-HMGA1 pathway may represent a new strategy to improve resistance of cancer cells to chemotherapy and IR treatment.

## Discussion

Previous studies have shown that HMGA1 performs oncogenic functions and promotes tumor progression by regulating autophagy, angiogenesis, epithelial-mesenchymal transition, cell cycle, and chemotherapy resistance (22, 26, 48, 49, 50). DNA damage is the main way in which most chemotherapeutic drugs kill tumor cells, and enhanced DNA damage repair ability is considered as an important cause of chemotherapy resistance. It has been reported that HMGA1 enhances DNA damage repair by promoting ATM and RAD51 expression, which leads to chemotherapy resistance (29, 50). In addition to DNA damage repair, coping with replication stress is another important characteristic for tumor cell survival. However, the role of HMGA1 in dealing with DNA replication stress remains elusive.

In this study, we revealed a novel mechanism by which HMGA1 promotes cell cycle progression and cell proliferation by maintaining DNA replication fork stability. HMGA1 recruits FANCD2 to protect stalled replication forks from degradation and assists with unwinding the R-loop, ultimately increasing the ability of tumor cells to cope with replication stress (Figure 1, Figure 2, Figure 3). Mechanistically, NUDT16 interacts with HMGA1 and they are uniformly distributed in all stages of the cell cycle. NUDT16 recognizes HMGA1 with PARylation at Glu 50 and reverse its PARylation, thereby preventing subsequent targeted degradation of HMGA1 by the E3 ubiquitin ligase CHFR (Figure 4, Figure 5, Figure 6). Notably, HMGA1 and NUDT16 were highly expressed in radioresistant cell lines, and targeting NUDT16-HMGA1 pathway significantly enhanced the sensitivity of tumor cells to chemotherapy and IR treatment (Fig. 7). Furthermore, HMGA1 was positively correlated with NUDT16 protein expression and negatively correlated with CHFR protein expression in HCC specimens (Fig. 8). These results suggest that targeting NUDT16-HMGA1 pathway is likely to become a new strategy to enhance the efficacy of chemotherapy and radiotherapy.

DNA replication is a highly efficient and precise process, but it often encounters replication obstacles. When the replication obstacles cannot be resolved quickly, the replication fork will stall, which in severe cases, results in DNA damage and cell death. Accumulating studies have shown that replication fork stability is closely related to cell survival and proliferation (12). Our data indicate that HMGA1 is recruited to stalled replication forks and protects them from degradation, preventing further DNA damage (Fig. 2). Moreover, HMGA1 interacted with FANCD2, and HMGA1 KO significantly reduced FANCD2 recruitment at stalled replication fork (Fig. 3, *A*–*C*). FANCD2 has been reported to inhibit nuclease DNA2 activity through its N-terminal domain. In addition, FANCD2 collaborates with RAD51 to inhibit the activity of multiple nucleases, including DNA2, MRE11, and EXO1 (21, 34, 35). Coincidentally, our data show that HMGA1 also interacts with RAD51 (Fig. S1*J*). Therefore, we speculated that HMGA1 most likely maintains stalled replication fork stability by recruiting FANCD2.

In addition to protecting stalled replication forks, timely resolution of replication stress is also critical. R-loop, the conflict between DNA replication and transcription, is one of the most important factors causing replication stress in tumor cells under high intensity of replication and transcription. Deregulation of R-loop is essential for the maintenance of DNA replication (51, 52). The FANCD2/FANCI complex has been reported to inhibit the formation of pathogenic R-loop by binding ssRNA and ssDNA species (53). In addition, the splicing factor SRSF1 mediates monoubiquitination of FANCD2, which promotes mRNA export and reduces the R-loop (36). Our results showed that R-loops were significantly increased after HMGA1 depletion, and the increased R-loops resulted in faster DNA strand degradation and more DNA damage (Fig. 3, *D–L*). Besides, HMGA1 also interacts with FANCI (Fig. S1*J*). This suggests that HMGA1 not only protects the stalled replication fork from degradation, but also alleviates replication stress result from R-loop by recruiting FANCD2.

Poly(ADP-ribosyl)ation (PARylation) is a type of protein posttranslational modification that regulates various biological processes, including cell metabolism, replication stress response, and DNA damage repair, by modulating the degradation and function of proteins (54, 55, 56). PARylation of substrates is a reversible process, while the reversal of PARylation is called dePARylation, which is mediated by some specific dePARylation enzymes, including PARG, NUDT9, NUDT16, MacroD1, MacroD2, ARH1, ARH3, TARG1, and ENPP1 (45, 57, 58). Our results showed that NUDT16 interacted with HMGA1, and that NUDT16 depletion significantly upregulated the PARylation and ubiquitination of HMGA1 and decreased its protein stability (Fig. 4), suggesting that the dePARylation of HMGA1 is mainly mediated by NUDT16. PARP1, PARP2, TNKS1, and TNKS2 have been shown to catalyze PARylation (39). Our data showed that PARP1 interacted with HMGA1, and knockdown of PARP1 greatly reduced PARylation of HMGA1 (Fig. 5, *A* and *B*). PARylation of HMGA1 is assumed to be mainly mediated by PARP1. We further found that the Glu 50 mutation of HMGA1 significantly reduced its PARylation and ubiquitination and enhanced its protein stability. Importantly, NUDT16 knockdown no longer affected HMGA1 PARylation and ubiquitination when Glu 50 was mutated (Fig. 5, *D* and *E*). It was clear that the major PARylation site of HMGA1 was Glu 50, and NUDT16 recognized PAR chains at Glu 50 of HMGA1. In summary, PARP1 acts as a “Writer” and NUDT16 as an “Eraser” to regulate HMGA1 PARylation homeostasis. Notably, it has been shown that NUDT16 regulates the protein stability of 53BP1 and CtIP *via* dePARylation. Deletion of NUDT16 not only reduced the protein levels of 53BP1 and CtIP, but also impaired their recruitment to DNA double-strand breaks (45, 59). This suggests that NUDT16, in addition to regulating HMGA1 protein degradation, most likely affects HMGA1 recruitment to stalled replication forks in a similar manner, which requires further experiments.

Several studies have shown that protein PARylation is closely related to ubiquitination (40, 41). PARylated proteins can be recognized by specific domains, including the WWE domain and the PBZ domain (56). For example, the WWE domain-containing E3 ubiquitin ligases RNF146, TRIP12, DTX1, DTX2, DTX4, and the PBZ domain-containing E3 ubiquitin ligases CHFR can recognize PARylated proteins and mediate their ubiquitination and subsequent protein degradation (60, 61, 62, 63). CHFR includes the fork head associated domain (FHA) at the N terminus, the RING domain (RF) in the middle, and the C terminus cysteine-rich region (CR), which contains the PBZ domain that recognizes PARylation (60). Our results show that HMGA1 recognition by CHFR depends on the PBZ domain of CHFR (Fig. 6). Notably, the loss of the RING domain also greatly reduced the affinity of CHFR for HMGA1. We speculate that the RING domain, which is responsible for transferring ubiquitin molecules, may somehow assist CHFR in derecognizing HMGA1. We also found that the mutation of Glu 50 of HMGA1 severely reduced the interaction between CHFR and HMGA1 (Fig. 6), suggesting that HMGA1 recognition by CHFR depends on HMGA1 PARylation. In summary, CHFR acts as a “Writer”, which recognizes and mediates the ubiquitination of PARylated HMGA1 through PBZ and RING domains.

In conclusion, our study revealed a novel mechanism by which HMGA1 promotes cell proliferation. HMGA1 promotes DNA replication and cell cycle progression by recruiting FANCD2, which reduces R-loop-induced replication stress and protects stalled replication forks from degradation. Furthermore, NUDT16 mediated dePARylation of HMGA1 to avoid the subsequent targeted degradation of HMGA1 by the E3 ubiquitin ligase CHFR and enhance its protein stability. Targeting the NUDT16-HMGA1 pathway exacerbated replication pressure and reduced the stability of stalled replication forks, ultimately improving the sensitivity of hepatocellular carcinoma cells to chemotherapy and IR treatment. This suggests that the NUDT16-HMGA1 pathway may be a novel target for enhancing the sensitivity of tumors to chemotherapy and radiotherapy in future clinical trials (Fig. 8*D*).

## Experimental procedures

### Cell culture and transfection

HeLa and HEK293T cells were derived from American Type Culture Collection (Manassas, VA, USA). HepG2 and HCC-LM3 cells were a gift from Prof. Hu (Sun Yat-Sen University Memorial Hospital, Guangzhou, China). LacO-LacI reporter cells (U2OS-265) were kindly provided by Dr Roger Greenberg (University of Pennsylvania, Pennsylvania). All cells were cultured in high glucose Dulbecco's modified Eagle's medium (Vigonob) containing 10% fetal bovine serum (Lonsera). Cells were cultured in a constant temperature incubator at 37 °C containing 5% CO_2_.

Cells were seeded in 6-well plates and subsequently transfected with Viafect (Promega) for plasmid and Lipofectamine RNAiMAX (Thermo Fisher Scientific) for siRNA according to the manufacturer's instructions. Cells were harvested 24, 48, and 72 h later or used for further experiments. The sequences of siRNAs used in this study are shown in Table S1.

### Antibodies and reagents

The antibodies and reagents used in this study are listed in Table S2.

### Plasmid construction and stable cell line establishment

The full-length complementary DNAs coding of HMGA1, RNaseH1, PARG, MacroD1/2, TARG1, ARH1/3, NUDT9/16, ENPP1, TNKS1/2, PARP1/2/3, RNF146, CHFR, TRIP12, and DTX1/2/4 were obtained from human HEK293T cells by PCR assay. And they were ligated to the pDONR221 vector and subsequently transferred to destination vectors with SFB tag (S protein, FLAG, and Streptavidin-binding peptide tags), hemagglutinin (HA) tag or GFP tag *via* Gateway Technology (Invitrogen) as described previously (64). HA-Ub-K6 (#22900), HA-Ub-K11 (#22901), HA-Ub-K27 (#22902), HA-Ub-K29 (#22903), HA-Ub-K33 (#17607), HA-Ub-K48 (#17605), and HA-Ub-K63 (#17606) were purchased from Addgene. Mutations of HMGA1 were constructed using the Takara MutanBEST Kit (Takara) and associated primers. The associated primers are listed in Table S3. All mutation sites were sequence verified.

HMGA1, NUDT16, PARP1, or CHFR CRISPR/Cas9 plasmids were constructed using pT2-CRISPRv2 vector and sgRNA. The primers of sgRNA are listed in Table S1. CRISPR/Cas9 plasmids were transiently transfected into HeLa cells and then treated with puromycin (1 μg/ml) for 48 h. After selection, the cells were digested, gradient diluted, and cultured to obtain monoclonal cells. Subsequently, monoclonal cells were collected for western blot verification, and finally KO cell lines of HMGA1, NUDT16, PARP1, or CHFR were obtained.

### Lentivirus packaging and infection

Tet-pLKO-puro was a gift from Dmitri Wiederschain (Addgene plasmid #21915). The shRNA sequences targeting NUDT16 were as follows: GCUACGCCAUA-CUGAUGCATT and confirmed by DNA sequencing. HEK293T cells were transfected with lentiviral vectors and packaged plasmids (pMD2G and pSPAX2) for 48 and 72 h, and the supernatant containing viral particles was collected. The cells were cultured in medium containing 8 μg/ml polybrene and virus particles for 12 h, and then treated with 2 μg/ml puromycin for 3 days to obtain stable cell lines. The cells were treated with 1 μg/ml doxycycline (Selleck) for 24 h to induce the expression of the above genes.

### Cell cycle synchronization

This procedure was performed as previously described (64). Briefly, HeLa cells were synchronized at the G1/S phase boundary by thymidine double blocking, and the cells were then replaced with fresh medium to continue growing. Cells cultured in fresh medium for 0 h (late G1 phase), 3 h (S phase), 6 h (G2 phase), and 9 h in the presence of nocodazole (100 ng/ml) (mitotic phase) were collected. The indicated cells were further analyzed by flow cytometry and western blotting.

### Immunofluorescence staining

Cells were seeded into confocal plates and subjected to plasmid transfection or IR treatment. Then cells were washed with PBS and fixed with 4% paraformaldehyde for 10 min, followed by permeabilization with buffer containing 0.5% Triton X-100 for 5 min at room temperature. After blocking with fluorescent blocking solution, cells were incubated with the primary antibodies at 4 °C overnight. After removal of the primary antibody, cells were washed three times with PBS and then incubated with the secondary antibody for 1 h at room temperature. Finally, cells were stained with 4′,6-diamidino-2-phenylindole for 10 min at room temperature to allow visualization of nuclear DNA, followed by fluorescence microscopy analysis.

### Proximity ligation assay

The indicated cells were pulse-labeled with 10 μM EdU for 15 min followed by treatment with 4 mM HU for another 3 h. After washing with PBS three times, the cells were pretreated with 0.5% Triton X-100 for 5 min at 4 °C followed by washing with PBS three times, and then fixed with 3% formaldehyde at room temperature for 15 min, washed with PBS three times and blocked with 3% bovine serum albumin at room temperature for 30 min. The resulting cells were subjected to Click-iT reaction to attach biotin to EdU and then incubated with the indicated primary antibodies at 4 °C overnight. PLAs were carried out by using a Duolink In Situ Red Starter Kit (DUO92101, Sigma-Aldrich) according to the manufacturer’s protocol. Finally, the images were acquired by Zeiss LSM 800 microscope and analyzed by ImageJ software.

### LacO-LacI system

U2OS-265 cells have 265 LacO repeats, which are specifically recognized by LacI, a DNA-binding protein. After mCherry-LacI-CHFR and GFP-HMGA1 mutant plasmids were constructed, U2OS-265 cells were transfected with the above-mentioned plasmids. After 24 h to 48 h, cells were washed, fixed, permeabilized, and blocked according to the immunofluorescence experimental procedures. Finally, the nuclear DNA was visualized with 4′,6-diamidino-2-phenylindole, and the cells were analyzed by fluorescence microscopy.

### DNA fiber spreading analysis

Cells were seeded into 6-well plates and then incubated with 25 μM ldU (Macklin, I811619) for 30 min. The cells were quickly washed with PBS for 3 to 4 times and then incubated with 250 μM CIdU (Sigma-Aldrich, C6891) for 30 min. After CIdU incubation, the cells were quickly washed with PBS for 3 to 4 times and then treated with 4 mM HU for an additional 5 h. After this, cells were digested and harvested by trypsin and suspended in PBS, and approximately 1000 cells were plated onto glass microscope slides with 8 μl lysis buffer (50 mM EDTA, 0.5% SDS, and 200 mM Tris–HCl). The slide was then tilted to allow the cell lysate suspension to slide slowly. The DNA fibers were fixed in 3:1 methanol: acetic acid for 20 min and denatured with 2.5 M HCl for another 30 min. After washing and blocking, the cells were incubated overnight with anti-IdU (OriGene, TA190129, 1:200) and anti-BrdU (Abcam, ab6326, 1:500) and stained with Alexa Fluor 488 goat anti-mouse (Thermo Fisher Scientific, A11001, 1:2000) and Alexa Fluor 555 goat anti-rat (Thermo Fisher Scientific, A21434, 1:2000) antibodies the following day. Finally, the images were acquired and analyzed by Zeiss LSM 800 microscope and ImageJ software.

### Alkaline comet assay

After IR treatment, cells were harvested and resuspended in PBS. Subsequently, 0.8% normal melting point agarose gel was first spread onto the slide. Then the cells were mixed with 0.6% low melting point agarose gel and placed on top of the 0.8% normal melting point agarose gel, covered with a cover slip, and left at 4 °C for 10 to 15 min. After gently removing the cover slips, the slides were lysed in lysis buffer (1.2 M NaCl, 100 mM Na_2_EDTA, 0.1% sodium lauroyl sarcosinate, and 0.26 M NaOH) for 30 min at 4 °C in the dark. The slides were then placed in an unwinding buffer (0.03 M NaOH and 2 mM Na_2_EDTA) at 4 °C in the dark for unwinding and electrophoresis. Subsequently, slides were washed with 0.4 M Tris buffer, stained with SYBR green DNA dyes (Thermo Fisher Scientific), and visualized and analyzed with fluorescence microscope and Comet Assay Software Project. Tail moment was used to evaluate the degree of DNA damage.

### Cell survival assay and cell proliferation assay

The cell survival fraction was determined by colony formation assay. In brief, HeLa and HCC-LM3 cells were seeded in 12-well plates (500 cells per well) and then treated with different concentrations of cisplatin for 24 h, or with different doses of IR, followed by continued culture for 2 weeks to form colonies. Colonies were finally fixed with 4% paraformaldehyde, stained with 0.1% crystal violet, and then photographed and counted.

Cell viability was detected by Cell Counting Kit-8 assay and EdU assay. Cells were seeded in 96-well plates, and absorbance values were measured at 450 nm on days 1, 3, and 5 during the culture according to the instructions. Cells were seeded in 12-well plates and incubated with EdU (10 μM) for 1 h, followed by washing, fixation, permeabilization, click reaction, as well as nuclear staining according to the kit instructions (Beyotime). Finally, the number of EdU-positive cells was observed by fluorescence microscopy.

### Cell cycle assay and apoptosis assay

Regarding the cell cycle experiments, cells were collected at different intervals after receiving the synchronization treatment. Then cells were resuspended in 70% cold ethanol and fixed overnight at −20 °C. The cells were washed once with PBS buffer, and mixed with staining buffer (50 μg/ml RNase, 25 μg/ml propidium iodide), and stained for 30 min at room temperature in the dark. Finally, the cell cycle distribution was detected by flow cytometry.

As for apoptosis experiments, cells were harvested after receiving transfection or IR treatment, and washed with PBS buffer. Cells were mixed with Annexin V/PI staining buffer (Bioscience) and stained for 15 min at room temperature in the dark. Finally, the cell apoptosis was detected by flow cytometry.

### Immunoblot and co-immunoprecipitation (co-IP) analyses

Co-immunoprecipitation and western blotting were performed as previously described (65). Briefly, cells were lysed with NETN buffer (20 mM Tris–HCl, 100 mM NaCl, 1 mM EDTA, and 0.5% Nonidet P-40) supplemented with protease inhibitors (Bimake) and benzonase nuclease (Merck Millipore) at 4 °C for 30 min, and then the cleavage products obtained after centrifugation were incubated with either indicated antibodies, anti-S beads (Merck Millipore) or anti-HA beads (Bimake) overnight at 4 °C. The next day, samples were washed three times with NETN buffer, resuspended in 1 × SDS loading buffer and boiled. Proteins were separated by SDS-PAGE and transferred to polyvinylidene fluoride membranes. Then blocking was performed in PBST buffer containing 5% milk, followed by incubation with the indicated antibodies. Finally, enhanced chemiluminescence western blot detection reagents were used to detect the relative protein content. Antibody information is listed in Table S2.

### RNA isolation and quantitative reverse transcription polymerase chain reaction analysis

RNA extraction and quantitative reverse transcription polymerase chain reaction were performed as previously described (65). In brief, after transfection or synchronization of cells, cellular RNA extraction, reverse transcription, and quantitative polymerase chain reaction were performed according to the manufacturer's instructions (EZBioscience). The primers used in this study are listed in Table S3.

### Immunohistochemistry

Dewaxing, hydration, antigen recovery and blocking of tissue chips were performed as previously described (65). After blocking, the tissue chips were incubated with the primary antibodies at 4 °C overnight. After removal of the primary antibody, tissue chips were washed three times with PBST and then incubated with the horse radish peroxidase-conjugated secondary antibody for 30 min at room temperature. After washing with PBS for three times, 3,3'-diaminobenzidine (DAB) staining was performed, followed by hematoxylin staining of the nucleus. Then, the tissue chips were dried and sealed with neutral resin. Finally, the images were acquired by Nikon Ni-U microscope and analyzed by Image-Pro Plus.

### Statistics

All statistical results are shown as the means ± SDs. Student’s *t* test or one-way ANOVA were performed with GraphPad Prism 8.0 software. Differences with *p* < 0.05 were considered to be statistically significant (∗∗∗*p* < 0.001; ∗∗*p* < 0.01; ∗*p* < 0.05; ns, not significant).

## Data availability

All data are available in the main text or the supplementary materials.

## Supporting information

This article contains supporting information.

## Conflict of interest

The authors declare that they have no conflicts of interest with the contents of this article.
