# Supplementary material for: NUDT16 enhances the resistance of cancer cells to DNA-damaging agents by regulating replication fork stability via reversing HMGA1 ADP-ribosylation
Source: J Biol Chem. 2025 Apr 25;301(6):108551. doi: 10.1016/j.jbc.2025.108551 (PMC12151214; doi:10.1016/j.jbc.2025.108551)
Supplement: Supplementary Material [file mmc1.docx]

**Supplementary Figure legends**

**Supplementary Figure 1**

(A) Analysis of HMGA1 protein levels in liver hepatocellular carcinoma (LIHC), lung adenocarcinoma (LUAD) and pancreatic adenocarcinoma (PAAD) in the Clinical Proteomic Tumor Analysis Consortium database (CPTAC) (http://ualcan.path.uab.edu/index.html). (B) The relationship between HMGA1 and survival of LIHC, LUAD, and PAAD was analyzed in the Gene Expression Profiling Interactive Analysis database (GEPIA2) (http://gepia2.cancer -pku.cn/index). (C-H) HMGA1 depletion significantly inhibited the proliferation of HeLa cells. Control and HMGA1-knockout cells were collected and lysed, and the protein expression of HMGA1 was detected by western blotting (C). Cell proliferation of control and HMGA1-knockout cells were detected by CCK-8 assay at day 1, day 3, and day 5 (n=3) (D), and by clonal formation assay (n=3) (E and F). EdU assay was used to detect the proportion of S phase cells in control and HMGA1-knockout cells. Scale bar, 10 μm. (n=3) (G and H). (I) After the synchronization treatment, cells at the indicated time points were collected and RNA was extracted to analyze HMGA1 mRNA levels in each cell cycle by qRT‒PCR. (J) After transient transfection with SFB-vector and SFB-HMGA1, the HeLa cells were collected and lysed for co-IP with anti-S beads and analysed by western blotting with indicated antibodies.

**Supplementary Figure 2**

(A) HepG2 and HeLa cells were collected and lysed for co-IP with anti-IgG or anti-HMGA1 antibodies and analysed by western blotting with the indicated antibodies. (B) HeLa cells were collected and lysed for denaturing co-IP with anti-IgG or anti-PAR antibodies and analysed by western blotting with the indicated antibodies. (C) After transient transfection with SFB-vector and SFB-NUDT16, the HEK293T cells were collected and lysed for co-IP with anti-S beads and analysed by western blotting with indicated antibodies. (D) Control and NUDT16-knockout cells were collected and lysed for western blotting with the indicated antibodies. (E) After HepG2 and HCC-LM3 cells were transfected with NUDT16 siRNA for 24 h, RNA was extracted from cells, and the mRNA levels of NUDT16 and HMGA1 were detected by qRT-PCR. (F) After synchronized treatment of control and NUDT16-knockout HeLa cells, cells at the indicated time points were collected and lysed，followed by western blotting with indicated antibodies. (G) Control and NUDT16-knockout cells were collected and lysed for co-IP with anti-IgG or anti-HMGA1 antibodies and analysed by western blotting with the indicated antibodies. (H) Control and NUDT16-knockout HeLa cells were transiently transfected with SFB-HMGA1. And then the cells were collected and lysed for co-IP with anti-S beads and analysed by western blotting with the indicated antibodies. (I) Control and NUDT16-knockout HeLa cells were transiently transfected with SFB-HMGA1, HA-tag ubiquitin (WT) and HA-tag ubiquitin mutants (K6, K11, K27, K29, K33, K48, K63). And then the cells were collected and lysed for co-IP with anti-HA beads and analysed by western blotting with the indicated antibodies.

**Supplementary Figure 3**

(A) HCC-LM3 cells were transfected with NUDT16 siRNAs for 48 h, and the NUDT16 protein levels were examined by western blotting analysis. (B-F) NUDT16 depletion significantly inhibited the proliferation of HeLa cells. Control and NUDT16-knockout cells were collected and lysed, and the protein expression of NUDT16 was detected by western blotting (B). Cell proliferation of control and NUDT16-knockout cells were detected by clonal formation assay (n=3) (C and D). EdU assay was used to detect the proportion of S phase cells in control and NUDT16-knockout cells. Scale bar, 10 μm. (n=3) (E and F). (G) Western blotting was used to detect the protein expression of HMGA1 and NUDT16 in HMGA1-knockout, NUDT16-knockout and NUDT16-HMGA1 double knockout HeLa cells. (H and I) Parental and 231RR cells with or without IR (8 Gy) treatment, the proportion of apoptotic cells was determined by Annexin V/PI staining and flow cytometry after 48 h. (J) 231RR cells were transfected with HMGA1 siRNA, and the protein expression of HMGA1 was detected by western blotting. (K) HCC-LM3 cells were transfected with HMGA1 siRNA or NUDT16 siRNA respectively, or co-transfected with HMGA1 and NUDT16 siRNA. And then the protein expression of HMGA1 and NUDT16 were detected by western blotting. (L-M) Inhibition of NUDT16-HMGA1 pathway enhances the efficacy of chemotherapy. Control, NUDT16-depleted, HMGA1-depleted, both HMGA1 and NUDT16-depleted HCC-LM3 cells were treated with different doses of cisplatin, and were continued in culture to form colonies. Colonies were quantified using ImageJ software.

**Supplementary Figure 4**

(A-C) Relative expression levels of HMGA1 (A), NUDT16 (B) and CHFR (C) proteins in samples of HCC patients with different pathological grades.


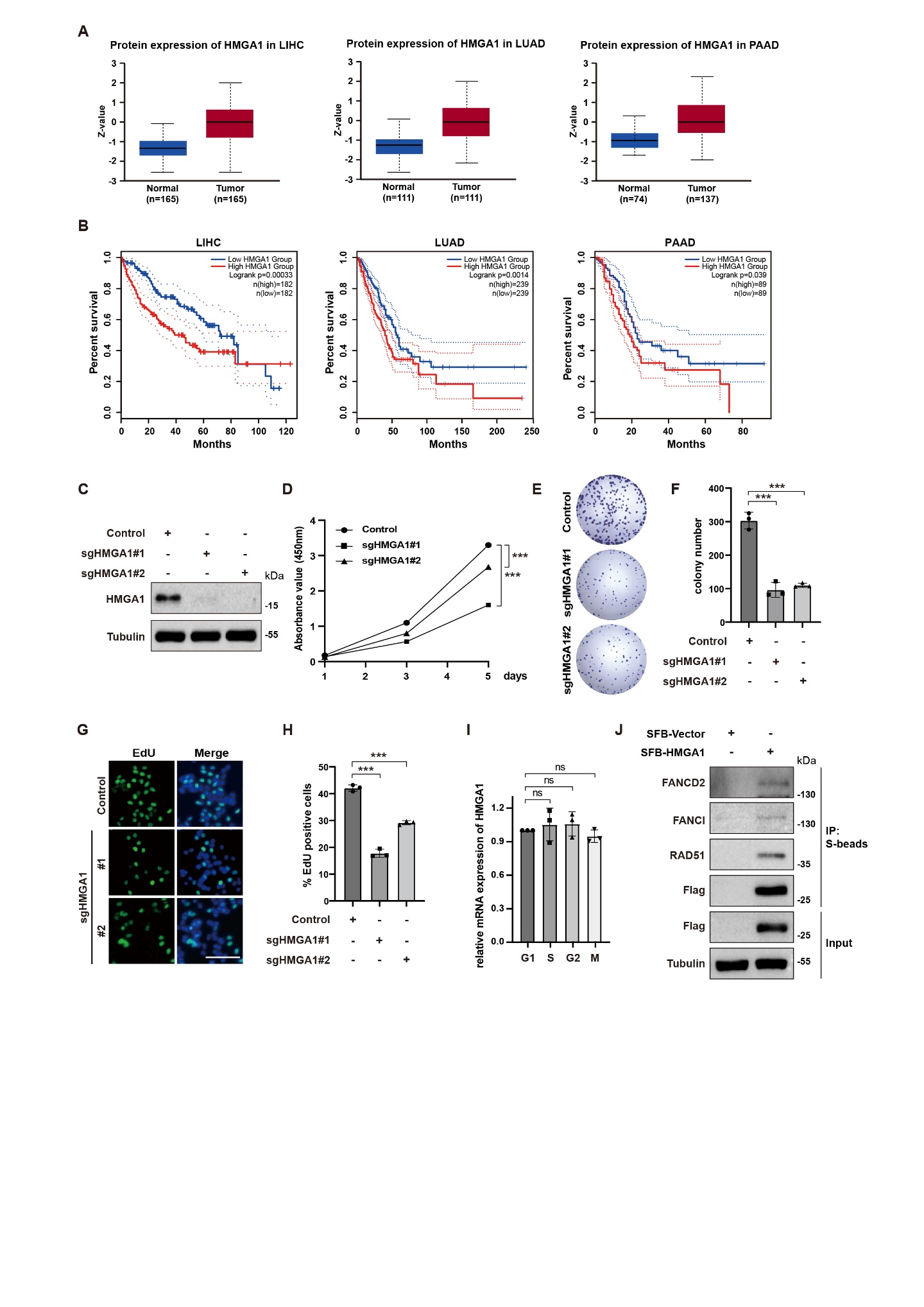
**Supplementary Figure 1**


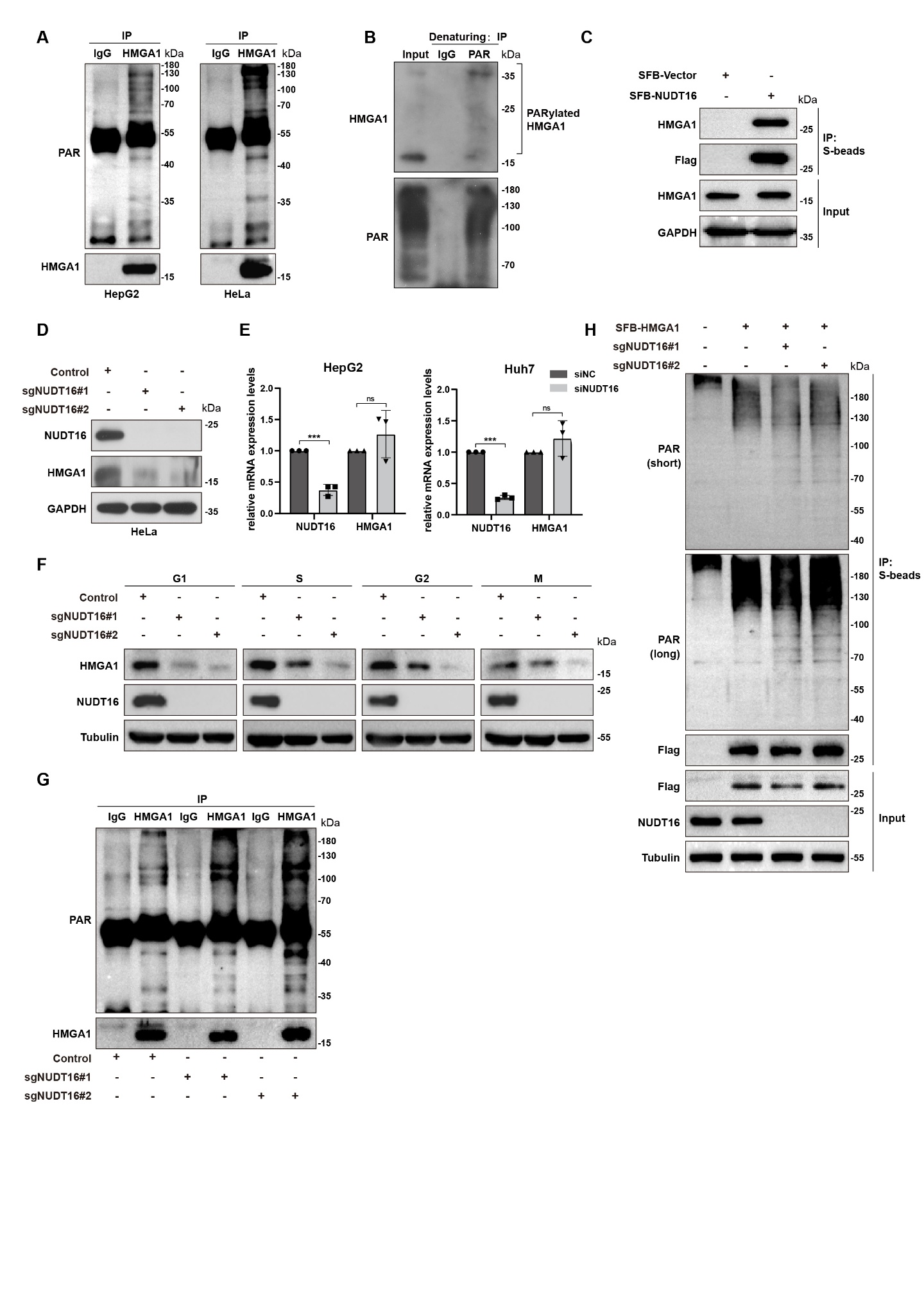
**Supplementary Figure 2**


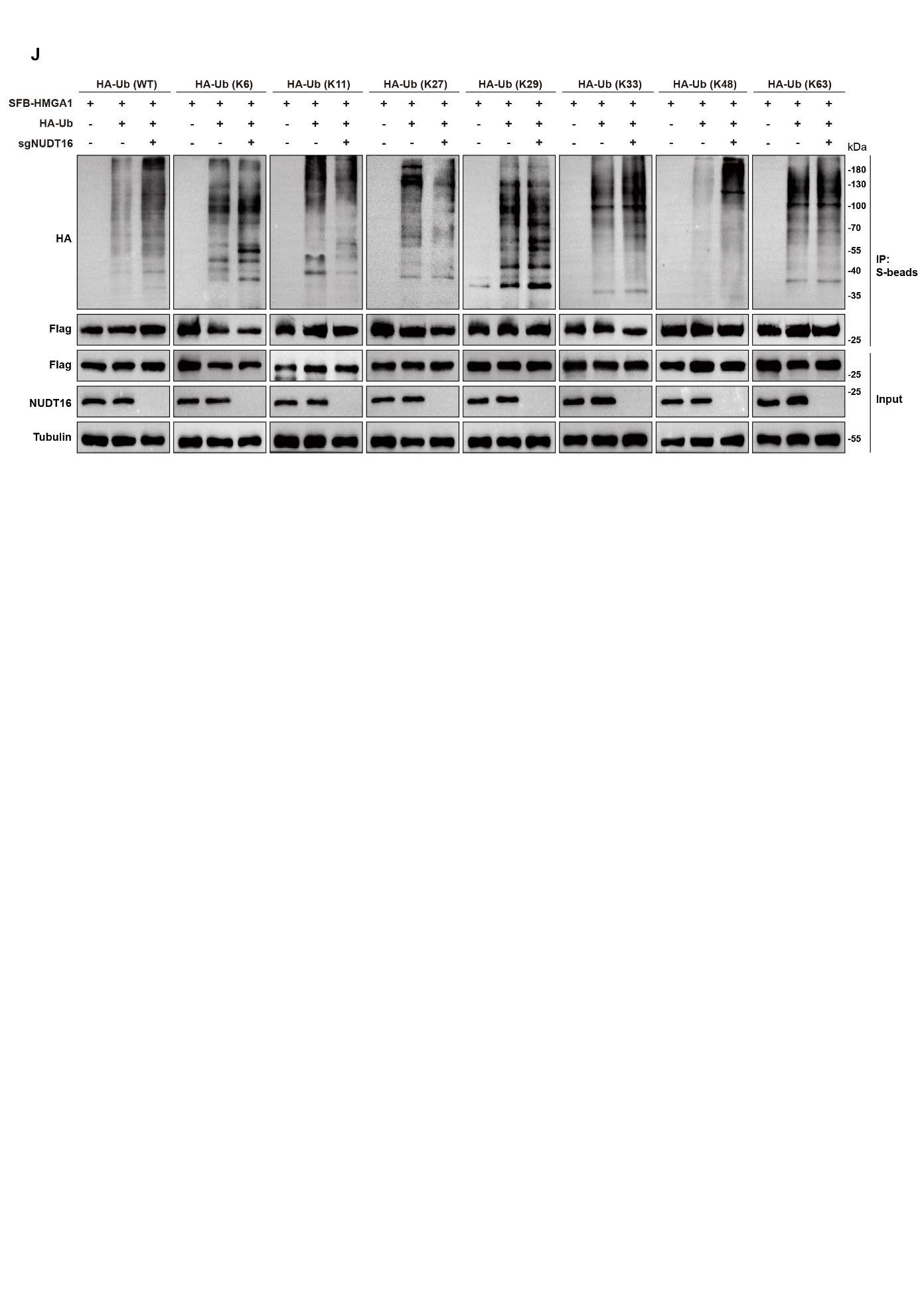


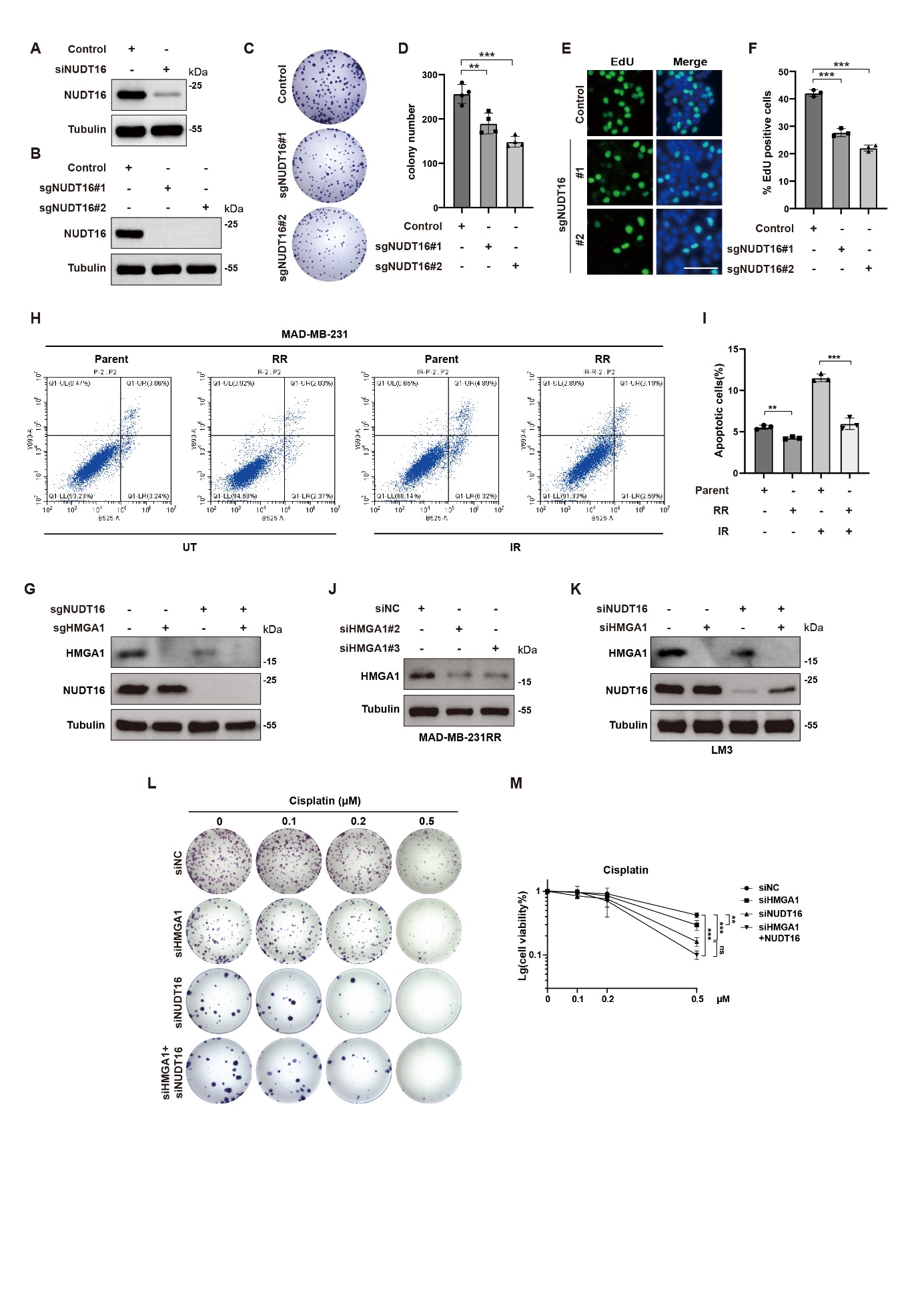
**Supplementary Figure 3**


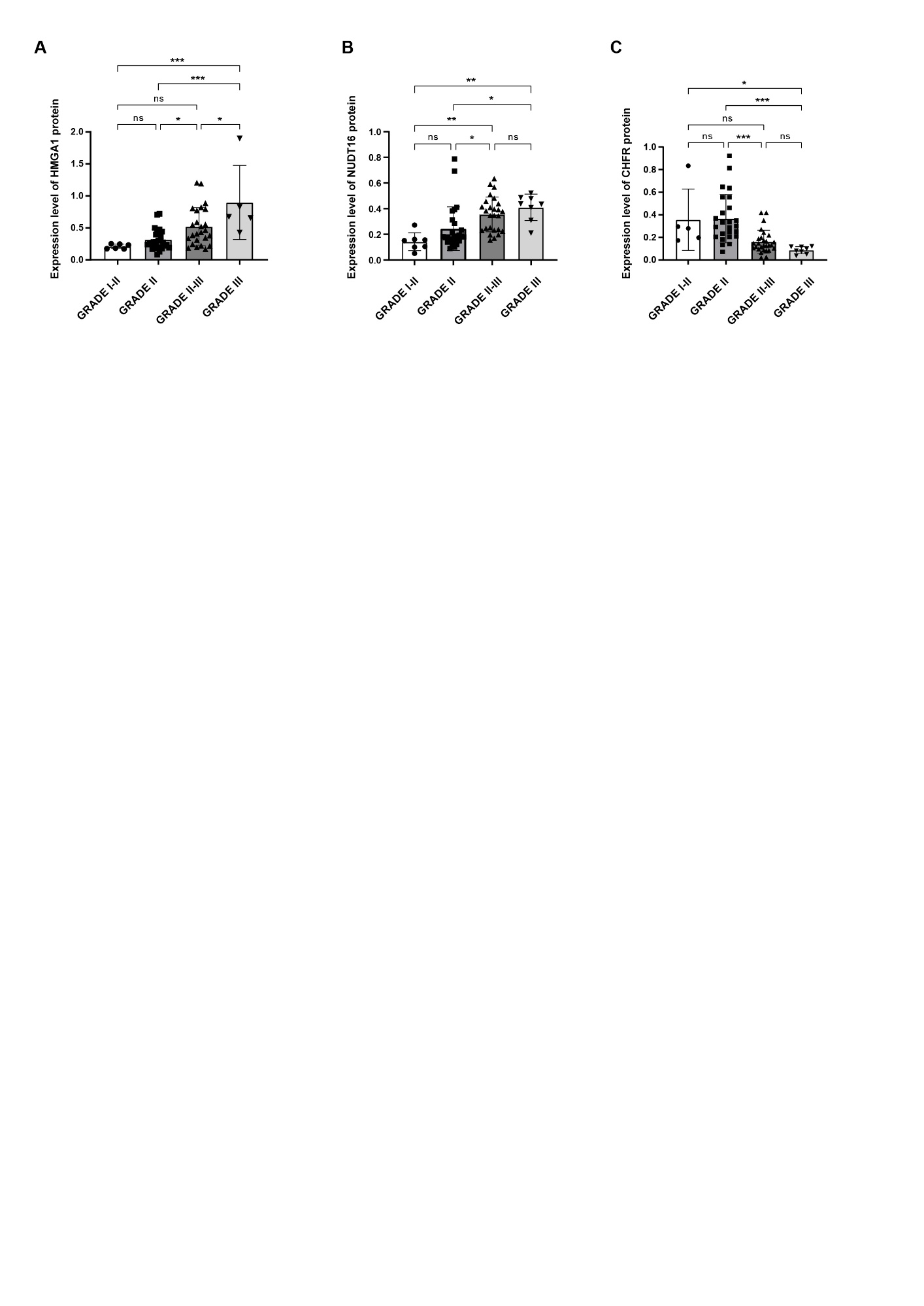
**Supplementary Figure 4**

**Supplementary Table 1 The sequences of siRNAs and sgRNA**

| **Genes** | **Source** | **Sequences (5’-3’)** |
| --- | --- | --- |
| siHMGA1#1 | IGE Biotechnology | 5’-CUCACCACCACACUACACA-3’ |
| siHMGA1#2 | IGE Biotechnology | 5’-GAGUACAUAUUGUGGUGAU-3’ |
| siHMGA1#3 | IGE Biotechnology | 5’-GCUGCUACCAGCGCCAAAU-3’ |
| siNUDT16 | IGE Biotechnology | 5’-GCUACGCCAUACUGAUGCATT-3’ |
| sgHMGA1#1 | IGE Biotechnology | 5’-AAAAGGACGGCACTGAGAAG-3’ |
| sgHMGA1#2 | IGE Biotechnology | 5’-AAGGACGGCACTGAGAAGCG-3’ |
| sgNUDT16#1 | IGE Biotechnology | 5’-AGGACAGAAGCCTAGAGGAC-3’ |

**Supplementary Table 2 Antibodies and reagents**

| **Antibody** | **Source** | **Identifier** |
| --- | --- | --- |
| HMGA1 | ABclonal | A4343 |
| NUDT16 | ABclonal | A17825 |
| CyclinB1 | Beyotime | AF1606 |
| CyclinE1 | Proteintech | 11554-1-AP |
| PARP1 | Proteintech | 22999-1-AP |
| CHFR | ABclonal | A10447 |
| FANCD2 | ABclonal | A2072 |
| FANCI | Proteintech | 20789-1-AP |
| RAD51 | Proteintech | 14961-1-AP |
| Flag | Beyotime | AF2852 |
| HA | ABclonal | AE008 |
| Ub | Cell Signaling Technology | #3936 |
| PAR | TREVIGEN | 4335-MC-100 |
| Tubulin | Santa Cruz | Sc-5274 |
| GAPDH | Signalway Antibody | #21612 |
| S9.6 | Kerafast | ENH001 |
| AF555-γH2AX | BD | 560446 |
| IdU | ORIGENE | TA190129 |
| CldU | abcam | Ab2326 |
| **Reagent** | **Source** | **Identifier** |
| cycloheximide | Selleck | S7418 |
| MG132 | Selleck | S2619 |
| HU | Sigma | H8627 |
| Cisplatin | Selleck | S1166 |
| BMN673 | Selleck | S7048 |
| doxycycline | Selleck | S5159 |

**Supplementary Table 3 The primers of HMGA1 and NUDT16**

| **Genes** | **Source** | **Sequences (5’-3’)** |
| --- | --- | --- |
| HMGA1-F | IGE Biotechnology | 5’-AGCGAAGTGCCAACACCTAAG-3’ |
| HMGA1-R | IGE Biotechnology | 5’-TGGTGGTTTTCCGGGTCTTG-3’ |
| NUDT16-F | IGE Biotechnology | 5’-CTGCGCTACGCCATACTGAT-3’ |
| NUDT16-R | IGE Biotechnology | 5’-GTCAGACGCTTGGCATAGAAG-3’ |
| HMGA1 (E47Q)-F | IGE Biotechnology | 5’-CAGCCCAGCGAAGTGCCAACAC-3’ |
| HMGA1 (E47Q)-R | IGE Biotechnology | 5’-CTTCTGACTCCCTACCAGCG-3’ |
| HMGA1 (E50Q)-F | IGE Biotechnology | 5’-CAAGTGCCAACACCTAAGAGAC-3’ |
| HMGA1 (E50Q)-R | IGE Biotechnology | 5’-GCTGGGCTCCTTCTGACTCC-3’ |
| HMGA1 (E47/50Q)-F | IGE Biotechnology | 5’-CAGCCCAGCCAAGTGCCAACACCTAAGAGAC-3’ |
| HMGA1 (E47/50Q)-R | IGE Biotechnology | 5’-CTTCTGACTCCCTACCAGCG-3’ |
